# Supplementary material for: Substrate colonization by an emulsion drop prior to spreading
Source: Nat Commun. 2021 Sep 30;12:5734. doi: 10.1038/s41467-021-26015-2 (PMC8484436; doi:10.1038/s41467-021-26015-2)
Supplement: Supplementary file 1 — Supplementary Information [file 41467_2021_26015_MOESM1_ESM.pdf]

Supplementary Discussion

“Substrate colonization by an emulsion drop prior to spreading”

Suraj Borkar and Arun Ramachandran

Contents

Supplementary Note 1. Ray tracing simulations for film shape reconstruction 2

Supplementary Note 2. Scaling equations based on Newtonian film drainage....7

Supplementary Note 3. Weissenberg numbers during film drainage....13

Supplementary Note 4. Cryo-SEM/Focussed Ion beam milling 15

Supplementary Note 5. Evidence to support the hypothesis of glycerol nucleation and growth....18

Supplementary Note 6. AFM images for surface roughness 22

Supplementary Note 7. Possibility of immobilized polymer layer near a polymer-glycerol interface....24

Supplementary Note 8. Effect of water on polymer confinement....32

Supplementary Note 9. Insights from past literature on the stability of nanobubbles and nanodroplets....34

Supplementary Note 10. Constancy of contact angle during island growth....41

Supplementary Note 11. Lower bound for length scale over which disjoining pressure is prevalent....43

Supplementary Note 12. Solubility measurement using Nuclear Magnetic Resonance (NMR) spectroscopy....47

Supplementary Note 13. Solubility measurement using confocal Raman spectroscopy....48

Supplementary Note 14. Estimation of viscosity of immobilized silicone oil layer 51

Supplementary Note 15. Concentration boundary layer near the drop interface 52

Supplementary References....54

Supplementary Note 1. Ray tracing simulations for film shape reconstruction

Ray tracing simulations provide an accurate means to reconstruct arbitrary film shapes by accounting for the curvature of the interface and the angular spread of incident light (Supplementary Fig. 1)[1–3]. Furthermore, this technique circumvents the problem of approximating the adjacent intensity extrema to reconstruct cosine branches based on the simple cosine theory[3–5]. Ray tracing can also be easily extended to more complicated optical systems. Here we have used the method of cubic splines to describe and deduce the shape of the films iteratively.

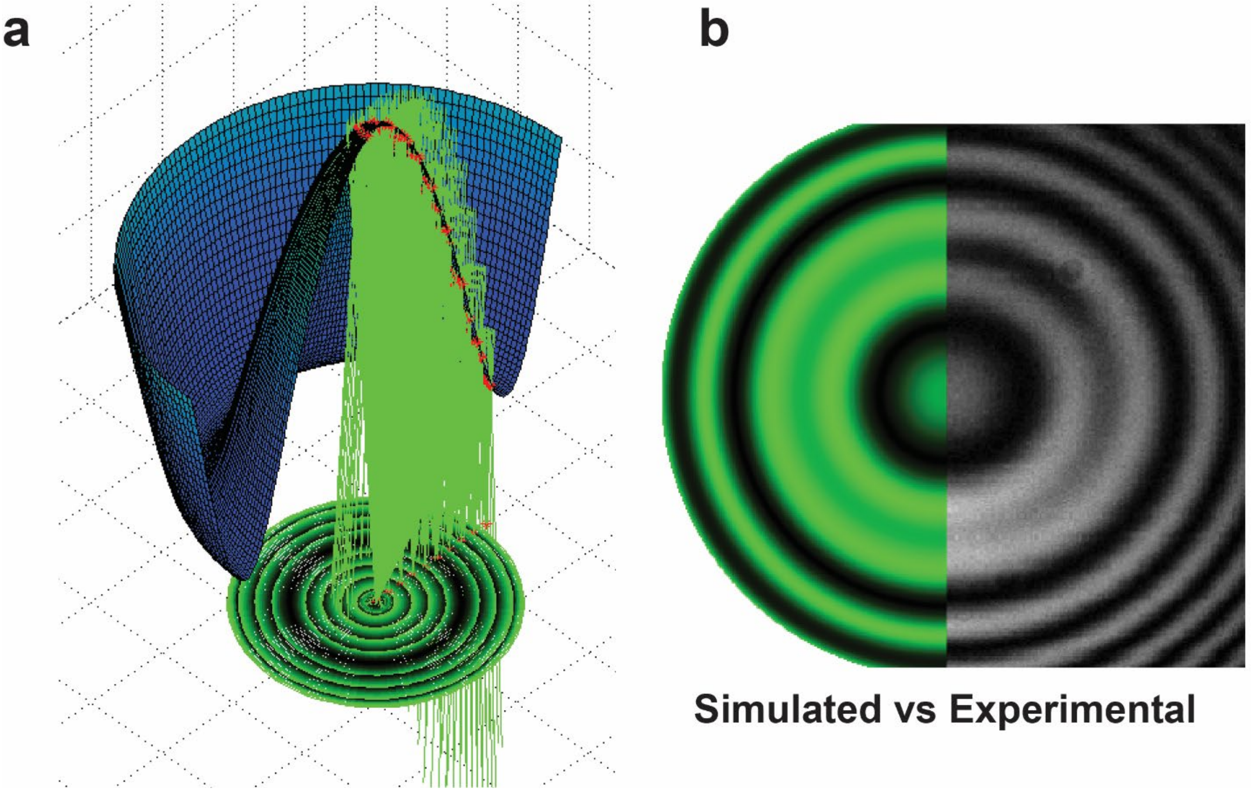

Supplementary Fig. 1. a) Ray tracing simulation of a dimpled film modeled using cubic splines, b) Comparison of simulated (left half) and experimental interference patterns (right half). Illumination wavelength: 549 nm

Firstly, the image background is subtracted to remove effects of inhomogeneous illumination[6]. The Hough transform is then applied to the images to deduce the center of symmetry of the

interference rings, and radially averaged intensities are extracted using this center[7]. Dual-wavelength interferometry was used to eliminate ambiguities in film heights and helped in the determination of absolute separation distances[4,5] (Supplementary Fig. 2). The evolution of the RICM fringes is observed and recorded as the droplet approached the mica sheet. A ray tracing algorithm previously described in the literature is then used to analyze the RICM fringes[1,2].

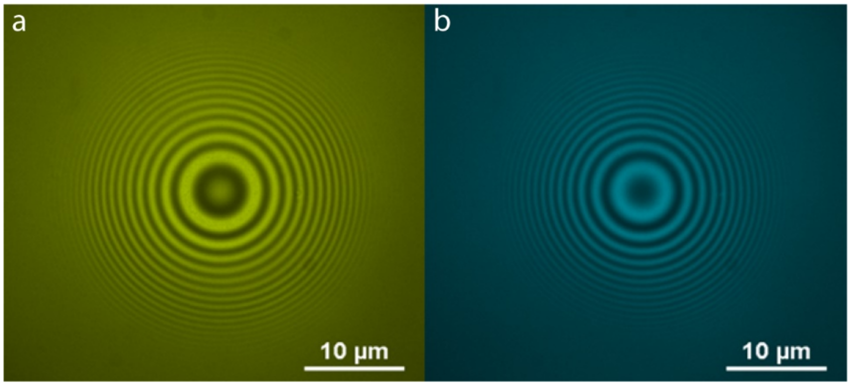

Supplementary Fig. 2. Interference pattern of a droplet under two different wavelengths of illumination: a) 549 nm, b) 485 nm

The ray tracing method involves simulating the local intensities at different points on the image plane by following the traces of ray backward, against the direction of propagation of light rays (Supplementary Fig. 3). The point intensity, $I_{refl}^{s,p}$, at a point B on the image plane, given by the contribution of intensities from all rays reaching B at all possible (θ, φ) is given by[1,2]

$$I_{refl}^{s,p} = \frac{\int_0^{2\pi} \int_0^{\alpha_{IA}} [R^{s,p}(\theta,\varphi)^* R^{s,p}(\theta,\varphi)] \left[I_0 / 2 \right] \sin\theta d\theta d\varphi}{\int_0^{2\pi} \int_0^{\alpha_{IA}} \sin\theta d\theta d\varphi}, \quad \text{(S1.1)}$$

where s and p denote the state of polarization with respect to the plane of polarization, I_0 is the incident light intensity, α_{IA} is the maximum illumination angle, and $R^{s,p}$ is the effective

reflection coefficient. $R^{s,p}$ depends on the path length difference Δ. The calculation of the optical path difference necessitates the use of a mathematical equation for the interface shape to locate the surface of reflection and define the normal.

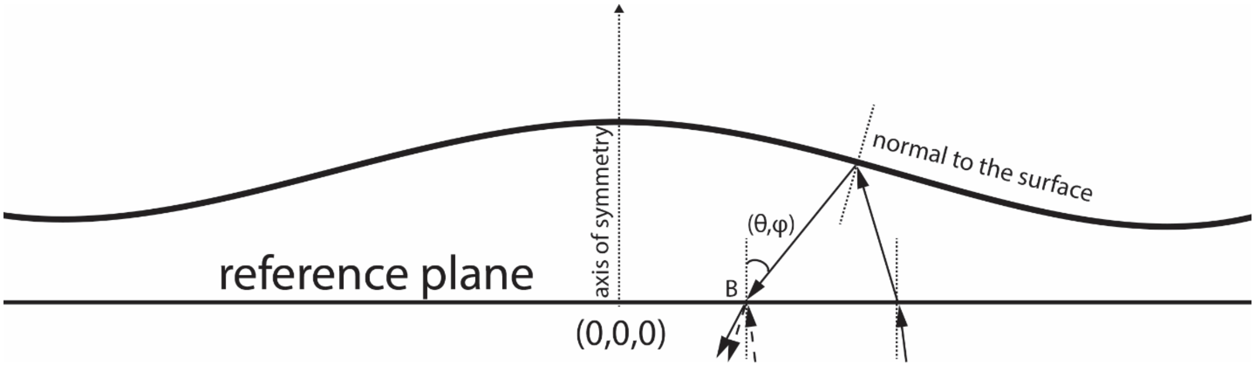

Supplementary Fig. 3. Ray intersections on an arbitrary shape

The film shape, $z = h(r)$, in the domain, $r \in [0, r_{\max}]$, is described using cubic splines by breaking it down into N piecewise cubic polynomials,

$$z = f_i(r) = a_i(r - r_i)^3 + b_i(r - r_i)^2 + c_i(r - r_i) + h_i \text{ for } r \in [r_i, r_{i+1}], \qquad \text{(S1.2)}$$

where the counter i varies from 1 to N (Supplementary Fig. 4). Note that $r_1 = 0$ and $r_{N+1} = r_{\max}$. To complete the definition of the spline (i.e. to obtain the coefficients a_i, b_i and c_i), we need to provide end boundary conditions[8]. For the axisymmetric shape considered in this work, the first derivative at $r = 0$ is set to zero and the not-a-knot condition is set at $r = r_{\max}$.

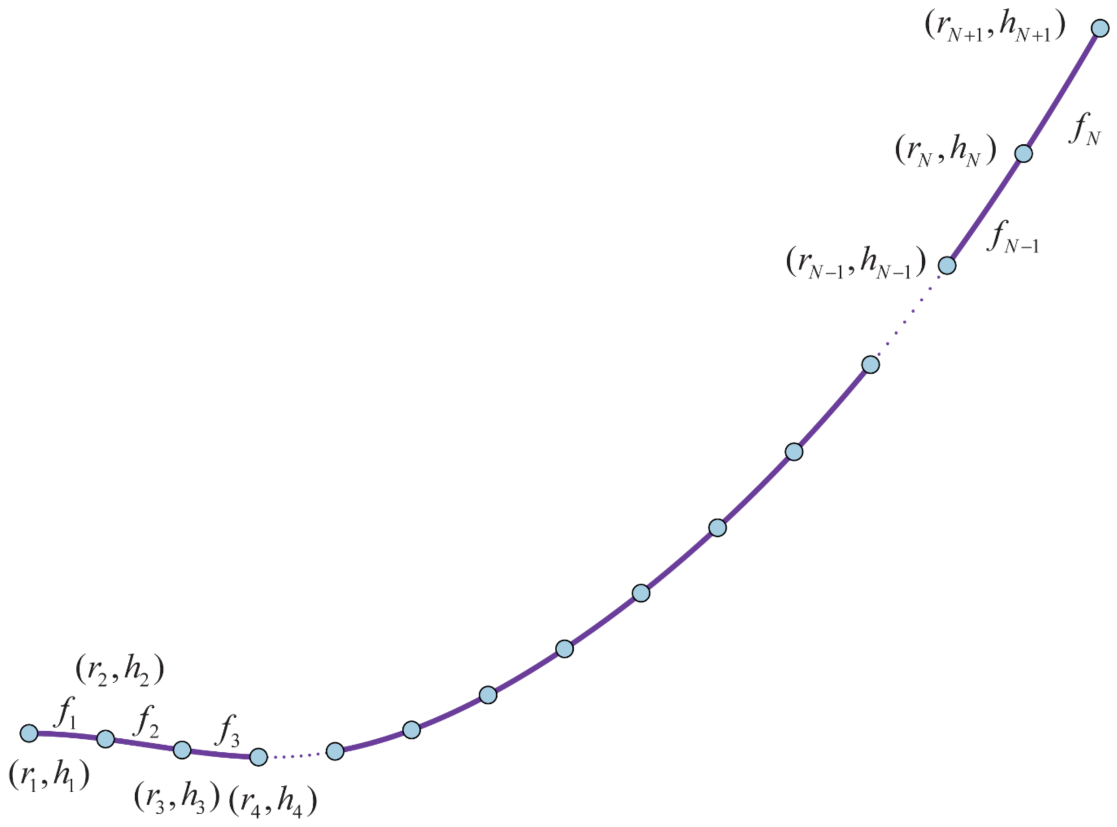

Supplementary Fig. 4. Piecewise cubic spline representation of a curve

The determination of the local intensity at any point or pixel requires the evaluation of the integration of the contribution of light rays reaching the point. This necessitates performing ray intersections with the spline curve in 3D space. For an axisymmetric film shape, the position vector corresponding to any point on the surface of the drop is given by

$$\overrightarrow{X} = r\cos\psi\,\hat{i} + r\sin\psi\,\hat{j} + h(r)\,\hat{k}, \tag{S1.3}$$

where r is the radial position, ψ is the azimuthal position relative to the center of symmetry and $h(r)$ is the fitted cubic spline. The normal to this spline surface can then be given by

$$\vec{n} = \frac{-\dfrac{dh}{dr}\cos\psi\,\hat{i} - \dfrac{dh}{dr}\sin\psi\,\hat{j} + \hat{k}}{\sqrt{\left(\dfrac{dh}{dr}\right)^2 + 1}}. \tag{S1.4}$$

Note that dh/dr is the first derivative of the fitted spline, which can be obtained by evaluating the first derivatives at each of the spline nodes and then splining the first derivatives at the nodes. The above two equations are necessary to evaluate the integrals for local intensity based on the effective reflection coefficients. Further details of the integration process are clearly described in reference [1,2]. The double integrals are evaluated using the MATLAB® function *integral2*.

By evaluating the point intensities at different points on the image plane, we can then compare the simulated intensity profile with the experimentally determined one to define a least squares error. The MATLAB® routine *fmincon* is employed to determine the film heights at the nodal positions of the spline that minimize the residual. The minimization algorithm needs an initial guess, and this is supplied by the simple cosine theory. By applying the principle of dual interferometry (Supplementary Fig. 2), we can determine the phases of the cosine branches to get an approximate film shape based on the simple cosine theory[4,5]. The computations are rendered fast by the vectorization of ray intersections with the spline surface as well as the parallelisation of the evaluation of point intensities. Also, the conversion of *ppval* to a C++ version (*ppuval*), for spline interpolation, leads to very fast computation (~5 s per radial intensity pattern).

Supplementary Note 2. Scaling equations based on Newtonian film drainage

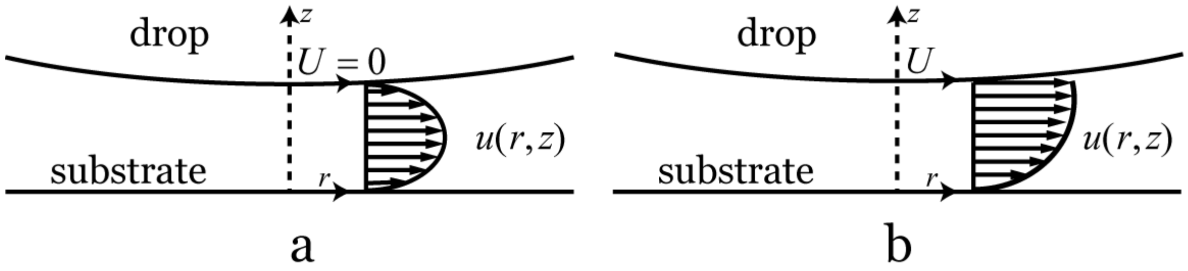

Supplementary Fig. 5. Velocity profile during drainage of lubricating film between a drop and a solid substrate when the drop interface is a) immobile (m<<1) and, b) fully mobile (m>>1).

The velocity profile in the thin film can vary between the case of a pure Poiseuille flow for large viscosity ratios (Supplementary Fig. 5a), to the case of a Couette-Poiseuille flow with a stress-free boundary condition for low viscosity ratios (Supplementary Fig. 5b). In general, we can write

$$u \sim u_t + u_p, \tag{S2.1}$$

where u_p is the Poiseuille contribution and u_t is the Couette contribution. A tangential stress balance at the interface yields

$$\frac{\hat{\mu} u_t}{a} \sim \frac{\mu\left(u_p - u_t\right)}{h}, \tag{S2.2}$$

which can be rearranged to obtain the following relationship between u_t and u_p.

$$u_t \sim \frac{m}{\left(m+1\right)} u_p, \tag{S2.3}$$

where m is the mobility ratio, defined as

$$m = \frac{a}{h} = \frac{1}{\lambda \varepsilon}, \tag{S2.4}$$

with $\varepsilon = h/a$, to distinguish between immobile and fully mobile interfaces. This ratio can range from very small values to very large values, depending on the viscosity ratio, the film size and the thin film height. For $m \ll 1$, the Couette contribution is much weaker than the Poiseuille contribution $\left(u_t << u_p\right)$ while for $m \gg 1$, the Couette and Poiseuille contributions are comparable $\left(u_t \sim u_p\right)$. The total flow in the film is, thus,

$$u \sim \left(\frac{m}{m+1}+1\right)u_p. \tag{S2.5}$$

It can be seen that while the viscosity ratio (through *m*) affects the actual magnitude of the total efflux velocity *u*, it does not affect the *scaling* of *u*, i.e. $u \sim u_p$ for both $m \ll 1$ and $m \gg 1$. In all of our scaling comparisons, the viscosity ratio was moderate (0.8 to 5) and lay in the limit $\varepsilon \ll \lambda \ll 1/\varepsilon$ identified by Yiantsios and Davis[9], i.e. we were always in the fully mobile limit in all the recorded data of *h* vs *t*. Therefore, even if the transition from an immobile to mobile regime occurred (which would lead to a dependence on the viscosity ratio), it had already manifested prior to the recording of the *h* vs *t* data. Thus, the drop viscosity does not feature in the scaling of the minimum height evolution with time. We note that the rate of drainage of a film between a solid surface and a drop can be quite different from the film between two drops of the same material. In the latter case, the Couette component can be significantly larger than the Poiseuille contribution[10] (e.g. see Ramachandran and Leal, 2016). This does not happen in the flow in the film between a drop and a surface.

The time dependence of the minimum film thickness can be derived by examining the rate of film drainage, taking into account the parabolic or Poiseuille component, which arises directly from the pressure gradient G pushing liquid out of the film, and the planar Couette component, which is the additional flow brought about by the mobile interface (Supplementary Fig. 5).

$$u = \frac{1}{2\mu} y(h-y)G + \frac{U}{h} y. \tag{S2.6}$$

A volume balance in the film gives

$$-\frac{dh}{dt} a^2 \sim u_{avg} ha, \tag{S2.7}$$

where u_{avg} is the average velocity in the film, and a is the radial length scale for flow. Substituting the expression for u_{avg} from equation (S2.7), we get

$$-\frac{dh}{dt} \sim \left(\frac{1}{12\mu} Gh^2 + \frac{U}{2} \right) \frac{h}{a}. \tag{S2.8}$$

The pressure gradient, G, scales as P/l_c, where P is the characteristic pressure difference between the film and the ambient suspending medium, and l_c is the length scale for the pressure gradient. The pressure gradient length scale, l_c, is $\sqrt{hR}$ for both spherical and dimpled films[11]. The film pressure and the radial length scale depend on the regime of drainage, as noted in Supplementary Table 1.

The drainage regimes can be better understood by inspecting how the lubrication pressure (P) compares with the Laplace pressure (σ/R). The transition height h_{trans} can be obtained by performing a balance between the lubrication pressure in the film and the Laplace pressure in the drop[9]

$$\text{Lubrication pressure} \sim \text{Laplace pressure} \tag{S2.9}$$

$$\frac{\Delta \rho g R^2}{h_{trans}} \sim \frac{\sigma}{R}, \tag{S2.10}$$

$$h_{trans} \sim \frac{\Delta\rho g R^3}{\sigma} = R\text{Bo}, \tag{S2.11}$$

where Bo is the Bond number, defined as $\text{Bo} = \frac{\Delta\rho g R^2}{\sigma}$. When $P \sim \sigma/R$ and the film is still spherical, a first transition height h_{trans_1} is observed, given by

$$h_{trans_1} \sim \mathcal{A} R\text{Bo}, \tag{S2.12}$$

where $\mathcal{A}$ is a constant. When the minimum film height reaches h_{trans_1}, the drainage dynamics changes in nature from a $h \sim e^{-t}$ to $h^{-1} \sim t$. Since the film is still spherical, the radial length scale (a) is still $\sqrt{hR}$. Further, a second transition in regime occurs at a transition height when the film shape changes from a spherical to a dimpled shape and is given by

$$h_{trans_2} \sim \mathcal{B} R\text{Bo}, \tag{S2.13}$$

where $\mathcal{B}$ is also a constant. This height corresponds to a change in scaling of h vs t from $h^{-1} \sim t$ to $h^{-3/2} \sim t$. The values of $\mathcal{A}$ and $\mathcal{B}$ were experimentally measured to be 4.2±0.6 and 0.7±0.1 approximately, respectively. The radial length scale (a) in the dimpled film regime is given by the film radius R_f.

A balance between the net buoyancy force and the capillary force provides the relationship between the film radius R_f and the drop radius[9],

$$\text{Net buoyancy force} \sim \text{Capillary force} \tag{S2.14}$$

$$\frac{4}{3}\pi R^3 \Delta\rho g \sim \frac{2\sigma}{R}\pi R_f^2, \tag{S2.15}$$

$$\text{or} \quad R_f \sim \left(\frac{2}{3}\frac{\Delta\rho g}{\sigma}\right)^{1/2} R^2 = R\left(\frac{2}{3}\text{Bo}\right)^{1/2}. \qquad \text{(S2.16)}$$

Supplementary Table 1 summarizes the film lubrication pressure and the radial length scale for different film drainage regimes. The h vs t scaling for different regimes can be by integrating the equation S2.8 with the definition of P and a in each regime.

Regime	Film pressure, P	Radial length scale, a	Minimum height, h
Spherical film $\left(P < \sigma/R\right)$	$\dfrac{\Delta\rho g R^2}{h}$	$\sqrt{hR}$	$h = h_0 \exp\left(-\dfrac{2c_1\Delta\rho g R t}{9\mu}\right)$
Spherical film $\left(P \sim \sigma/R\right)$	$\dfrac{\sigma}{R}$	$\sqrt{hR}$	$h^{-1} = h_{trans_1}^{-1} + c_2\dfrac{\sigma}{\mu R^2}t$
Dimpled film $\left(P \sim \sigma/R\right)$	$\dfrac{\sigma}{R}$	$R\text{Bo}^{1/2}$	$h^{-3/2} = h_{trans_2}^{-3/2} + c_3\dfrac{\sigma}{\mu R^{5/2}\text{Bo}^{1/2}}t$

Supplementary Table 1. The film pressure, radial length scale and the minimum film height arising from scaling arguments in the different film drainage regimes. The parameters c_1, c_2 and c_3 are constant scalars that emerge from the scaling analysis.

System	R (μm)	c_1 (exponential regime)	c_2 (spherical regime)	c_3 (dimpled regime)
SO1000-G-PS	31	1.39 (1.386,1.395)		
	45	1.94 (1.919,1.951)		
	47	1.3 (1.3,1.305)		
	52	1.23 (1.233,1.238)		
	80	1.23 (1.221,1.23)		
	93	1.03 (1.028,1.032)	0.049 (0.048 ,0.051)	
	106	0.83 (0.83,0.835)	0.0481 (0.048, 0.049)	
	106 (2nd)	0.99 (0.992,0.997)	0.0551 (0.054 ,0.056)	
	112	1.46 (1.455,1.465)	0.119 (0.115,0.123)	
	116	0.99 (0.981,0.989)	0.0618 (0.061,0.063)	
	133	0.99 (0.987,1.002)	0.0644 (0.06,0.069)	
	136	1.1 (1.09,1.108)	0.074 (0.068, 0.08)	
	142	0.92 (0.912,0.923)	0.0645 (0.063,0.066)	
	142 (2nd)	1.13 (1.125,1.143	0.096 (0.092,0.1)	
	195		0.0659 (0.064,0.068)	0.45 (0.444,0.459)
	240		0.0988 (0.097,0.101)	0.25 (0.238,0.253)
SO1000-G-M	40	1.13 (1.122,1.13)	0.161 (0.156,0.165)	
	66	1.22 (1.218,1.225)	0.089 (0.088,0.089)	0.42 (0.398,0.447)
	70	0.95 (0.947,0.954)	0.0801 (0.0798, 0.0804)	0.35 (0.344,0.3545)
	74	1.27 (1.268,1.273)	0.209 (0.206,0.212)	0.44 (0.435,0.451)
	115	0.89 (0.885,0.891)	0.153 (0.152,0.154)	1.47 (1.464,1.485)
	119	1.05 (1.036,1.057)	0.083 (0.082,0.085)	0.6 (0.592,0.602)
SO500-G-M	61	0.87 (0.868,0.873)	0.059 (0.058,0.06)	
	71	1.06 (1.062,1.068)	0.168 (0.163,0.173)	0.51 (0.496, 0.530)
	83	0.9 (0.903,0.907)	0.072 (0.07,0.073)	0.20 (0.198,0.21)
	104	0.95 (0.943,0.951)	0.074 (0.072,0.076)	0.31 (0.303,0.308)
	127	0.89 (0.878,0.896)	0.071 (0.069, 0.073)	0.34 (0.328,0.352)
	140	1.33 (1.183,1.476)	0.107 (0.099,0.115)	1.98 (1.845,2.124)
SO1000-G-NS	28	0.97 (0.966,0.978)		
	35	1.04 (1.035,1.043)		
	81	1.11 (1.03,1.11)	0.115 (0.107,0.124)	0.63 (0.587, 0.667)
	112	2.12 (2.103,2.138)	0.197 (0.192,0.203)	0.65 (0.605,0.699)
	140		0.118 (0.111,0.125)	0.54 (0.476, 0.611)
	169		0.29 (0.286,0.294)	0.67 (0.615,0.720)
PO-SO500-M	35	0.88 (0.876,0.88)	0.084 (0.08,0.087)	
	53	1.1 (1.099,1.106)	0.154 (0.138,0.17)	
	67	1 (0.995,1.023)	0.077 (0.074,0.08)	0.87 (0.5548,1.195)
	90	1.06 (0.983,1.136)	0.071 (0.068,0.075)	0.75 (0.689,0.806)

Regime not observed due to incoherence of light when h>1 μm

Regime not observed due to film instability or immobilized polymer films

Supplementary Table 2. Table shows the experimental values of prefactors c_1, c_2 and c_3 for all the liquid combinations and substrates employed. The numbers in the brackets in each entry in the table are the values including 95% confidence intervals.

Supplementary Note 3. Weissenberg numbers during film drainage

It is well known that polymeric melts can exhibit elastic, non-Newtonian effects when subjected to a flow. The applied stress causing the polymer to flow is also capable of extending the polymer chains, which can result in non-Newtonian effects[12]. In this section, we show that non-Newtonian effects can be ignored in the drainage of the polymer films. To demonstrate this, we calculate a dimensionless quantity called the Weissenberg number , given by[12]

$$\mathrm{Wi} = \dot{\gamma}\lambda_r, \tag{S3.1}$$

where $\dot{\gamma}$ is the characteristic shear rate in the film and λ_r is the relaxation time of the polymer chain. If $\mathrm{Wi} << 1$, the flow in the film can be assumed to be Newtonian. Non-Newtonian behaviour is manifested only when $\mathrm{Wi} \sim 1$ or $\mathrm{Wi} >> 1$.

Let us first estimate the shear rate during the film drainage process. During the initial stages of the film drainage of the polymer melt, the film is spherical, and the shear rate in the film can be shown to scale as[13]

$$\dot{\gamma} \sim \frac{\Delta\rho g R^{3/2}}{\mu h^{1/2}}. \tag{S3.2}$$

One can see that the shear rate is inversely related to the film thickness h. As h decreases, the shear rate increases. But as discussed in the previous section, as the film becomes thinner, the pressure in the film also rises. When the film pressure scales as the Laplace pressure, the film undergoes flattening and dimpling. For a dimpled film, the shear rate in the film scales as[13]

$$\dot{\gamma} \sim \frac{h^{1/2}\sigma}{\mu R^{3/2}}. \tag{S3.3}$$

One can see that in this limit, the shear rate in the film decreases as the film thins out. Thus, the shear rate shows a maximum during the film drainage process, with the maximum occurring

when the film pressure scales as the Laplace pressure. This occurs at the transition height h_{trans}, which we have shown to scale as $R\,\mathrm{Bo}$ (Fig. 2c,e). Substituting $h_{trans} \sim R\,\mathrm{Bo}$ in equation (S3.2), we get the maximum shear rate in the film during the drainage process as

$$\dot{\gamma}_{\max} \sim \frac{\Delta\rho^{1/2} g^{1/2} \sigma^{1/2}}{\mu}. \quad \text{(S3.4)}$$

Interestingly, this is independent of the drop radius. Taking $\Delta\rho = 290\ \mathrm{kg/m^3}$, for glycerol-silicone oil system, $\mu = 1000\ \mathrm{cP}$ and $\sigma = 30\ \mathrm{mN/m}$, we get $\dot{\gamma}_{\max} \sim 10\ \mathrm{s^{-1}}$.

Out of the two silicone oils we have used, the 1000 cP silicone oil has the higher relaxation time, equal to $\lambda_r \sim\ 1\ \mathrm{ms}$ [14]. Therefore, the maximum Weissenberg number for film drainage can be estimated to be $\mathrm{Wi} = \dot{\gamma}_{\max}\lambda_r = 0.01$, which is weak. Thus, any polymer stretching and relaxation effects induced by bulk shear can be ignored in our experiments, and the bulk fluid motion may be regarded as Newtonian.

Supplementary Note 4. Cryo-SEM/Focussed Ion beam milling

Cryogenic scanning electron microscopy (cryo-SEM) coupled with focussed ion beam milling (FIB) has been gaining popularity as a means to directly image nanoscale topological features at liquid/liquid and liquid/solid interfaces[15,16]. This section describes the protocol we developed to prepare specimen samples for the purpose of imaging the nucleated glycerol islands that are formed on mica underneath glycerol parent drops embedded in a cured silicone oil medium.

Mica sheets were sputter-coated with gold on one side. The other side was cleaved with scotch tape until a thickness of less than 10 μm was obtained. Sylgard 184 (Dow Corning) PDMS precursor was mixed with curing agent (10:1 ratio) and degassed to remove air bubbles using a vacuum pump connected to a desiccator. The mixture was then poured on to the mica sheet, gold-coated side down, to create a thin layer. Drops of glycerol were created and allowed to settle under gravity. The sample was then allowed to cure under ambient conditions for 24 hrs (Supplementary Fig. 6 a). This was then mounted mica side up, on a ½ inch diameter SEM stub (Supplementary Fig. 6 b,c). Conductive paint was applied on the edges and allowed to set overnight. The SEM stubs were then placed under a stereomicroscope to mark the drop positions using a Sharpie® marker (Supplementary Fig. 6 d). This allowed the ready determination of the drop location during FIB milling, as mica is not SEM-transparent.

The SEM stubs were introduced into SEM chamber of a Zeiss® NVision 40 cryo-SEM/FIB instrument, which uses gallium ions for micromilling. The SEM chamber was evacuated to ensure that no moisture deposition was caused. The temperature of the sample was lowered down to -120° using liquid N_2 cooling. A trench, 120 μm wide and 20 μm deep (Supplementary Fig. 6 e,f), was milled at the Sharpie® marked spots from the mica side of the mica/polymer interface, and not the polymer. This was because of the low thickness of mica side, purposely controlled by us to be less than 10 μm. The polymer side needed to be as thick as the glycerol drop diameter (~400 μm) in order to encase the drop. Therefore, milling from the polymer side would have

required trenches that were significantly deeper and wider than milling from the mica side, and this would have greatly prolonged the milling process due to the slow milling rates.

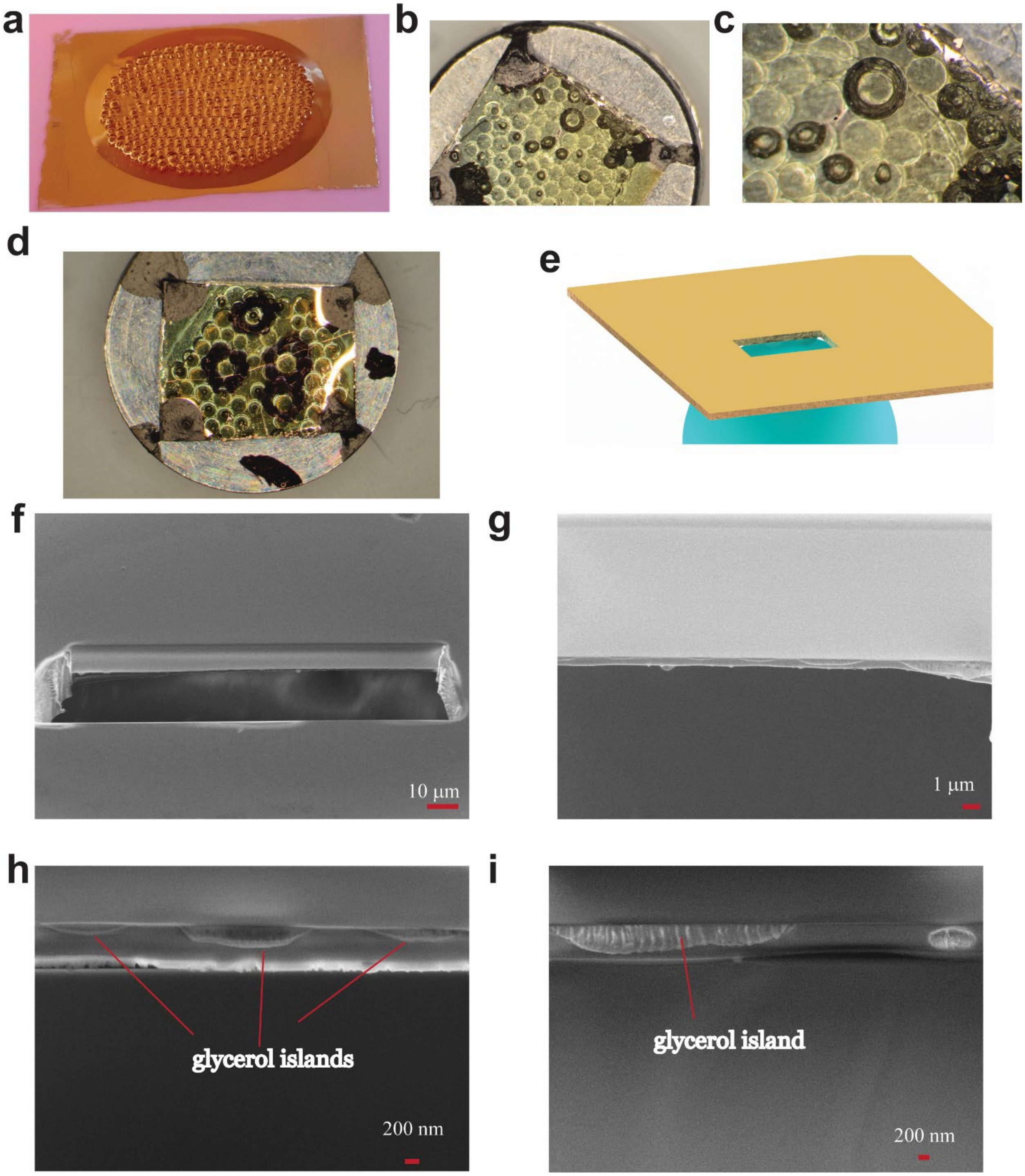

Supplementary Fig. 6. a) Glycerol droplets embedded in cured PDMS with gold coated mica substrate, b,c) Sample mounted on a ½ inch SEM stub with mica facing up, d) Drop locations marked using a Sharpie® for determination of area of interest for milling during FIB/SEM, e) Schematic to demonstrate the viewing perspective of SEM images, f) Milled trench in the Sharpie®-marked area. This area corresponds to region of about 120 μm across from the drop center location, g,h,i) SEM images showing nucleated sites under the parent drop.

Supplementary Note 5. Evidence to support the hypothesis of glycerol nucleation and growth

Our hypothesis that the glycerol drop is responsible for the formation of islands is corroborated by noting that islands nucleate and grow only underneath the parent drop. Supplementary Movie 2 shows an experiment where the field of view was gradually moved laterally (in the r direction, see Fig. 1a) along the mica surface and away from the contact zone between a drop of glycerol and mica. The video was captured by employing both brightfield in transmission mode and RICM in reflected mode. The brightfield illumination was used to demarcate the drop edge, with the help of diffraction patterns, for a constricted field diaphragm. The video clearly shows that the islands are present only in the vicinity of the contact zone.

To further validate the hypothesis, we realized that silicone oil presaturated with glycerol should also display similar nucleation and growth of glycerol islands, even in the absence of a parent glycerol drop. Silicone oil was presaturated with glycerol by mixing silicone oil and glycerol in a volume ratio of 30:1. The emulsion was allowed to equilibrate for 24 hours with periodic mixing in between. This was later centrifuged using a Sorvall Legend Z1 centrifuge (Thermo Scientific®) with a swinging bucket rotor at 4700g for 1 hour. The excess undissolved glycerol moves to the bottom of the centrifuge tube, leaving a silicone oil column at the top saturated with glycerol.

This presaturated silicone oil was then poured over the mica and plasma treated SU-8 substrates. As can be see in the figure below (Supplementary Fig. 7), RICM revealed the nucleation and growth of glycerol islands on these substrates, thus confirming the diffusion-mediated transport and nucleation of glycerol on to the mica surface and plasma-treated SU8.

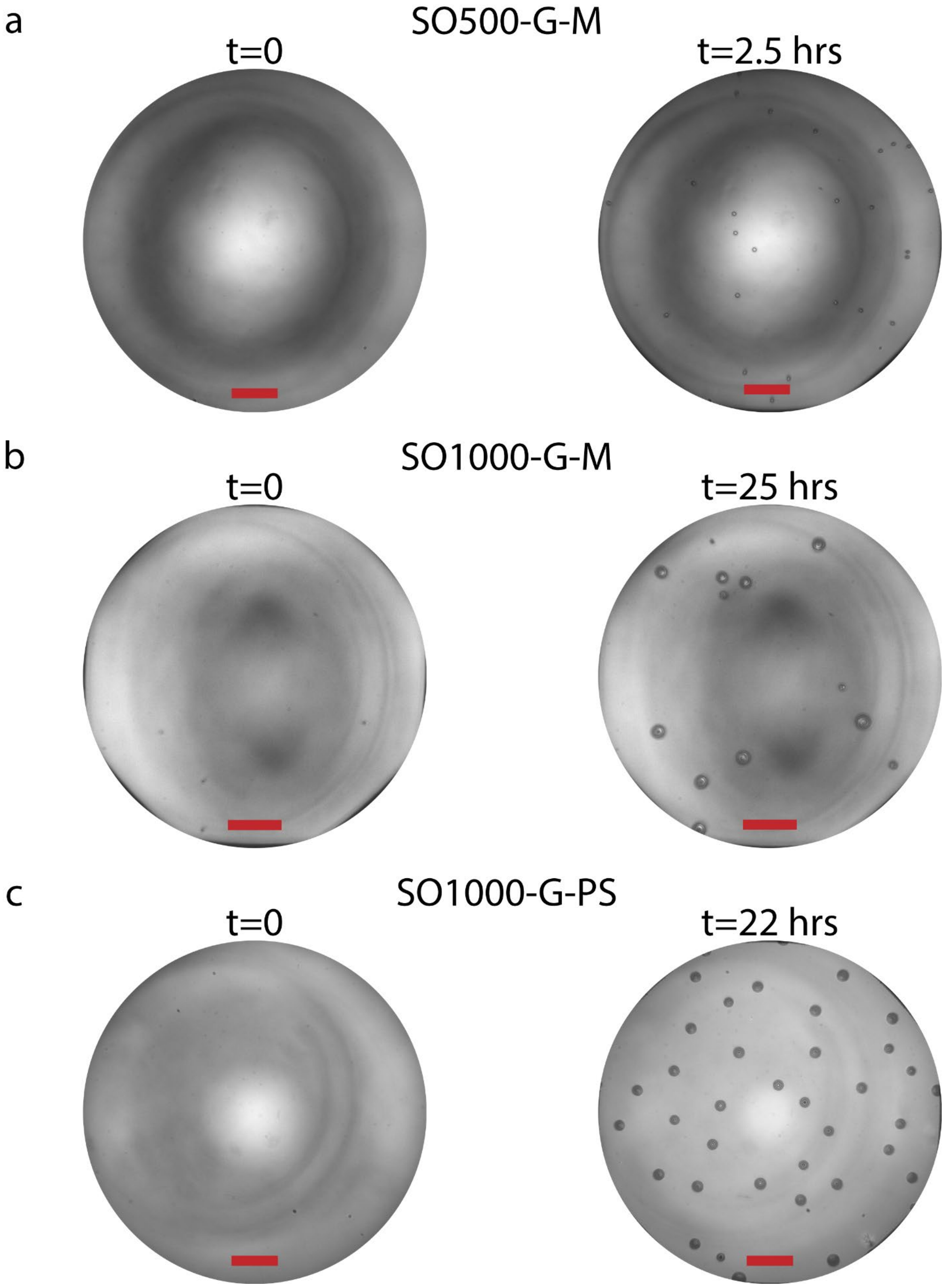
a
SO500-G-M
t=0
t=2.5 hrs
b
SO1000-G-M
t=0
t=25 hrs
c
SO1000-G-PS
t=0
t=22 hrs

Supplementary Fig. 7. Nucleation tests conducted for pre-saturated silicone oil a) SO 500/mica, b) SO 1000/mica, c) SO 1000/plasma-treated SU8. Illumination wavelength: 549 nm. Scale bar: 20 μm

Is water present in the island?

While the exact composition of these islands is not known to us, it can be proved the islands are largely composed of glycerol. Glycerol being a hygroscopic liquid, we determined the amount of water present in the glycerol used in the experiments reported in this work. A sensitive measure of the percentage of water in concentrated aqueous glycerol solutions is the viscosity of the mixture. For the determination of water content, a Carri-med rheometer (TA instruments, CSL2 500k) was used in the cone-and-plate configuration. Supplementary Fig. 8 shows that the viscosity of the glycerol mixture is 850 cP at 18 °C, which suggests the presence of 4 % water by volume[17].

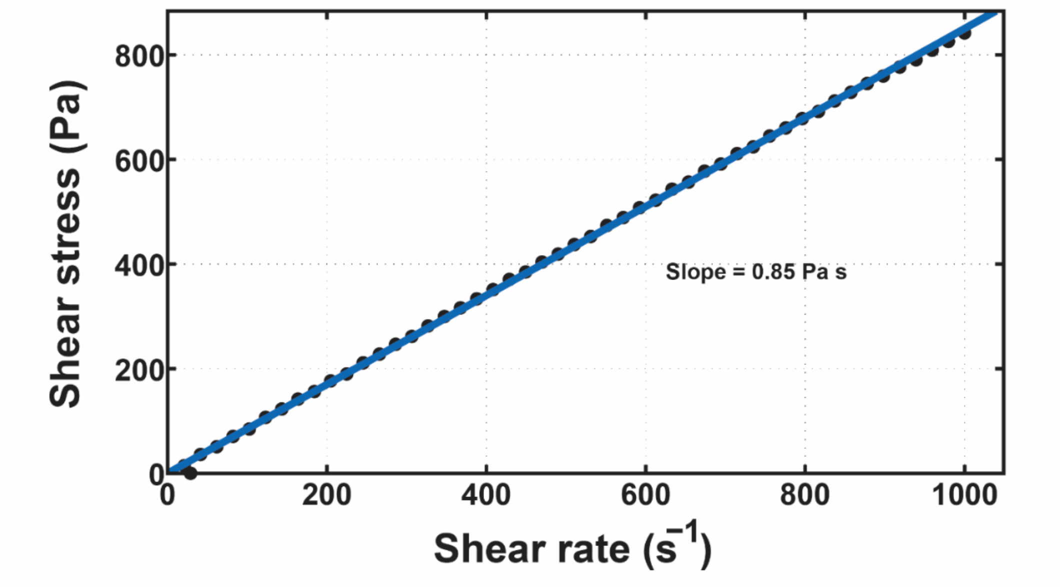

Supplementary Fig. 8. Shear stress vs. shear rate plot to obtain the viscosity and hence, the moisture content in glycerol.

Water has a lower solubility in SO than glycerol, but being a smaller molecule, its diffusivity is expected to be larger. It is not obvious, therefore, whether the nucleated phase would contain a greater or a lower percentage of water than the parent drop. But, since the appearance of these phases is in the form of dark fringe patches, it can only be a liquid phase whose refractive index is higher than that of silicone oil[4] (n =1.403), implying that the islands definitely contain more than 50% glycerol, the composition at which glycerol-water mixture is refractive index-matched with SO[18].

Supplementary Note 6. AFM images for surface roughness

SU8 is a UV-curable epoxy resin used in standard soft lithography techniques. Here, we use SU8 spin-coated on a glass coverslip, followed by UV-curing, as a substrate to study the effect of wettability on the nucleation and growth of islands underneath a parent drop. We use the term 'native SU8' when the cured polymer resin is used without any further treatment. Native SU8 yields a contact angle between glycerol/silicone oil/mica of 89°, due to the non-polar nature of SU8. When the cured SU8 is plasma-treated before use, the contact angle drops to about 55°, rendering it more water-wetting. This is due to an increase in polar groups such as C=O and COO[19]. Furthermore, plasma-treatment also leads to local flow of the polymer resin, causing increased surface roughness[19] and hence, more conducive to the nucleation and growth of islands. In this section, we conduct AFM measurements in tapping mode to measure the r.m.s surface roughness of native SU8 and plasma-treated SU8 (Supplementary Fig. 9). A Bruker® BioScope Resolve AFM microscope was used with a cantilever of spring constant of 6 N/m (resonant frequency 320 kHz) in tapping mode. Plasma treatment was performed using a hand-held corona treater (BD-20AC, Electro-technic Products®). SU2075 was the SU8 polymer resin used, which was spin-coated at 4000 rpm onto a No.0 borosilicate glass coverslip (Thermo Scientific®) to yield a 60 µm thick layer of SU8.

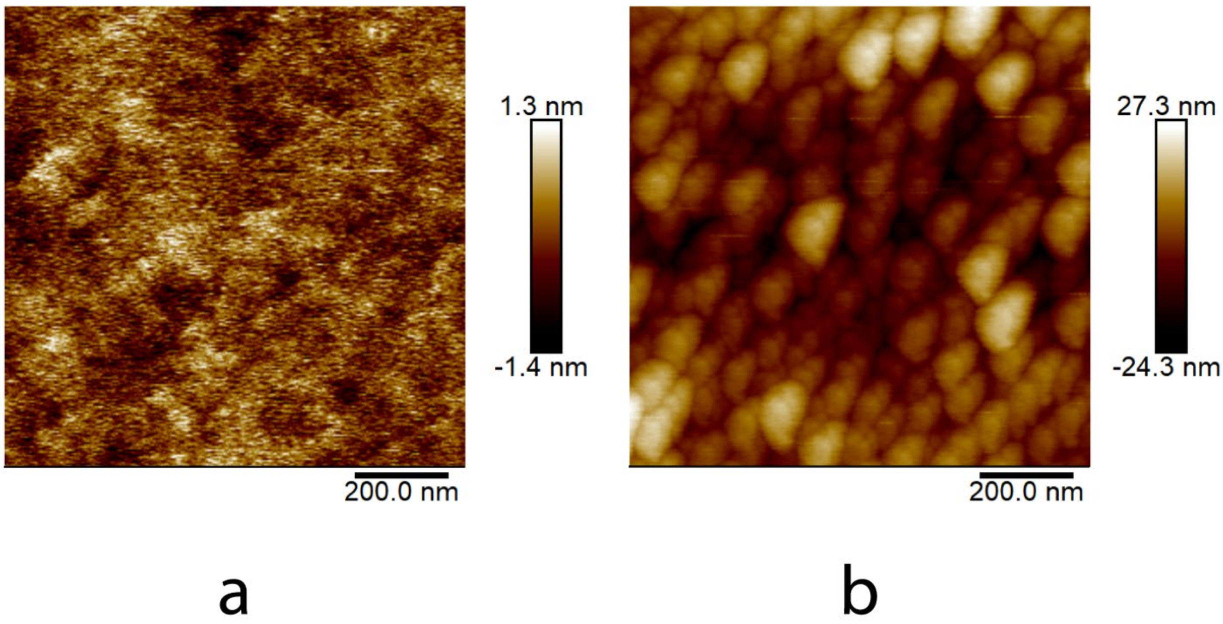

Supplementary Fig. 9. AFM images showing surface roughness in a) native SU8, b) plasma-treated SU8. The r.m.s surface roughness for native SU8 and plasma-treated SU8 is 0.379 nm and 7.44 nm, respectively.

Supplementary Note 7. Possibility of immobilized polymer layer near a polymer-glycerol interface

This section discusses the possible reasons for the observation of delayed coalescence of the parent drop with the nucleated islands. This is an essential aspect of this work due to the fact that the underlying unsubstantiated mechanism is the reason for the discovery of this new wetting phenomena. We majorly explore the possibilities of interfacial charging, and thin film polymer structuring and immobilization.

There has been a considerable amount of work done to show that a pristine water/hydrophobic interface (hydrophobic being oil or even air) can be negatively charged due to a selective adsorption of hydroxyl groups. The origin of free dangling hydroxyl groups was attributed to the fact that interfacial water molecules have broken hydrogen bonds at a 2D surface such as a liquid-liquid interface rather than in the bulk[20–22]. A more recent understanding of this interfacial charging is that rather than adsorption of hydroxyl groups due to self-dissociation of water molecules, a charge transfer mechanism involving an imbalance of accepting and donating hydrogen bonds leads to an effective negative charge at a water/hydrophobic material interface[23,24]. Yaminsky et al. (2010)[25], showed using a thin film balance setup that an air/water interface can be naturally charged with a charge potential of -57 mV with a Debye length of 152 nm. The electric double layer (EDL) forces were sufficient to stabilize thick water films of the order of 100 nm. Addition of small amounts of electrolytes decreased the Debye length by screening this residual charge at the interface, causing film instability. Similarly, Ivanov and Kralchevsky (1997)[26], Karraker and Radke (2002)[27], Leunissen et al. (2007)[28] and Creux et al. (2009)[29] showed evidence of negative charge at an oil-water interface due to the difference in the dielectric constants of the two phases. The zeta-potential was measured to be of $\mathcal{O}$(-100 mV) with a strong pH dependence. The zeta potential dropped above zero for lower values of pH.

The zeta-potential at silicone oil-water interface was also measured by Gu and Li (1998)[30] and was reported to vary between -40 mV at neutral pH to +25 mV at a pH of 3. No measurements were found in literature for silicone oil-glycerol interfaces. While we would not expect this interface to be charged (glycerol cannot self-dissociate; pKa=14.4 [31]), the glycerol employed in the experiments does have trace amounts of water which could potentially charge the interface. It is not clear, though, whether water would preferentially arrange at a glycerol-silicone oil interface, leading to electrostatic repulsion that would hinder coalescence.

To test the possibility of water dissociation at the interface leading to interfacial charging and hindrance of coalescence, we conducted experiments with water drops settling in dehydrated SO1000 towards a plasma-treated SU8 substrate. Gu and Li (1998)[30] showed that the isoelectric point for the water-silicone oil interface is at a pH of 5. If electrostatic repulsion is indeed the dominant mechanism for inhibiting coalescence, then performing experiments at the isoelectric point would mitigate the repulsion and lead to faster coalescence. In our experiments, we prepared pH 5 water by the addition of HCl. Supplementary Movie 6 shows the RICM images of a pH 5 water drop settling under gravity towards a SO1000-plasma-treated SU8 substrate. As may be seen, there is nucleation and growth of water islands upon the approach of the drop, and the coalescence of the parent drop with the islands did not take place, even when the growing islands pushed the parent drop up, against gravity. This suggests that strong electrostatic repulsion caused by the segregation of hydroxyl ions and/or by a charge transfer mechanism at the silicone oil-aqueous interface are not likely responsible for hindering coalescence. Furthermore, our experiments with glycerol drop-castor oil combination, did not show delayed coalescence, which leads us to believe that the polymeric nature of the suspending medium plays a key role in the inhibition of coalescence [see Supplementary Movie 5 (pristine castor oil) and Supplementary Fig. 10 (dehydrated castor oil)].

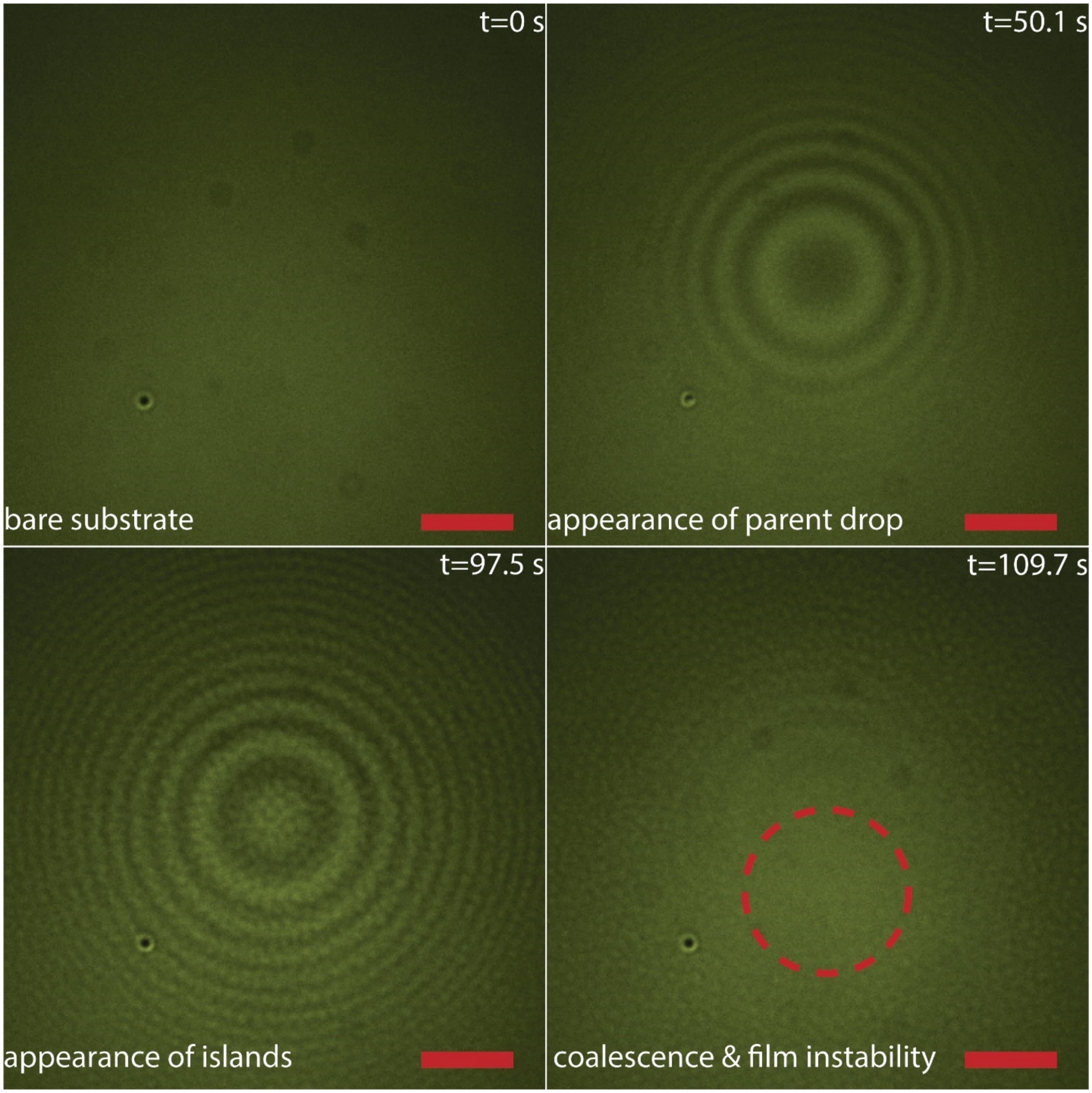

Supplementary Fig. 10. a) Glycerol drop settling in dehydrated castor oil, towards a plasma-treated SU8 substrate, coalesces with the nucleated islands and spreads instantly. Scale bar: 5 μm

We now discuss what we consider to be the more likely reason for the inhibition of coalescence – polymer immobilization at the glycerol – silicone oil interface. The phenomenon is manifested in the form of delayed coalescence leading to extended periods of island growth underneath the parent drop. We hypothesize that immobilization of a polymer film is possible at a liquid/polymer

melt interface. While there is adequate literature on polymer film immobilization at polymer melt/solid interface[32–34] , to the best of our knowledge, no studies have been performed to investigate this behavior at a liquid/liquid interface. Our first clue is the strong effect of the presence of trace amounts of water in silicone oil. As may be seen in Supplementary Movie 7 and as also in Supplementary Note 8, the presence of dissolved water in the polymer melt prior to its introduction on the surface leads to instantaneous coalescence of the parent drop with the interfacial islands. This behaviour has been recorded in literature for a polymer melt/solid interface[35,36], and was attributed to the disruption of the immobilization and structuring of the polymer chains.

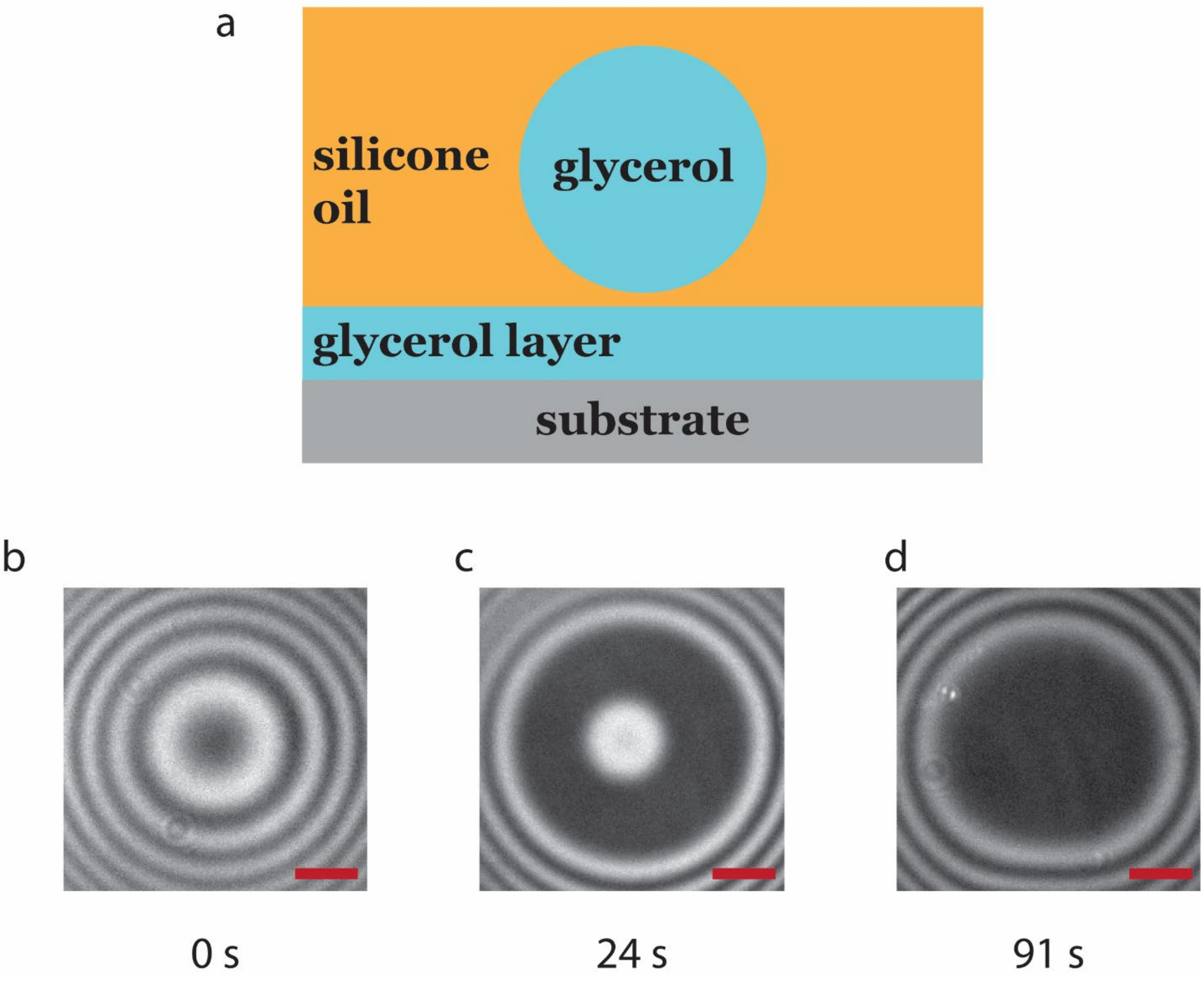

Supplementary Fig. 11. a) Experiments were conducted wherein a glycerol drop $(R = 83\ \mu\text{m})$ settled towards a thin glycerol film to show evidence of polymer confinement at a liquid/liquid interface. b),c) and d) show evolution and formation of a steady film of silicone oil (SO1000), whose film thickness is close to 10 nm. Scale bar: 2 μm

To show the substrate has no effect on the observation of hindered coalescence, we conducted experiments of glycerol drops settling towards a thick glycerol layer spin-coated on a solid substrate. A glycerol layer of thickness less than 40 μm was spun-coated onto a mica substrate, which was glued to a No.0 glass coverslip. This was to ensure the imaging plane was at the glycerol film/silicone oil interface, and to enable the capture of the interference patterns of a settling glycerol drop towards the glycerol layer. We observe drainage of the silicone oil film between the glycerol drop and glycerol layer, similar to that at a mica or SU8 interface, with the formation of a steady film of silicone oil (SO1000) of thickness 10 nm (Supplementary Fig. 11. a) Experiments were conducted wherein a glycerol drop $(R = 83\ \mu\text{m})$ settled towards a thin glycerol film to show evidence of polymer confinement at a liquid/liquid interface. b),c) and d) show evolution and formation of a steady film of silicone oil (SO1000), whose film thickness is close to 10 nm. Scale bar: 2 μm). The fact that coalescence is inhibited, suggests a repulsive disjoining pressure in the film.

A second observation is that when islands exhibit contact angles greater than 90° due to substantial pinning on a plasma-treated SU8, neighbouring islands do not coalesce instantly when they come into contact (Supplementary Fig. 12). Instead, a steady film is observed between adjoining islands.

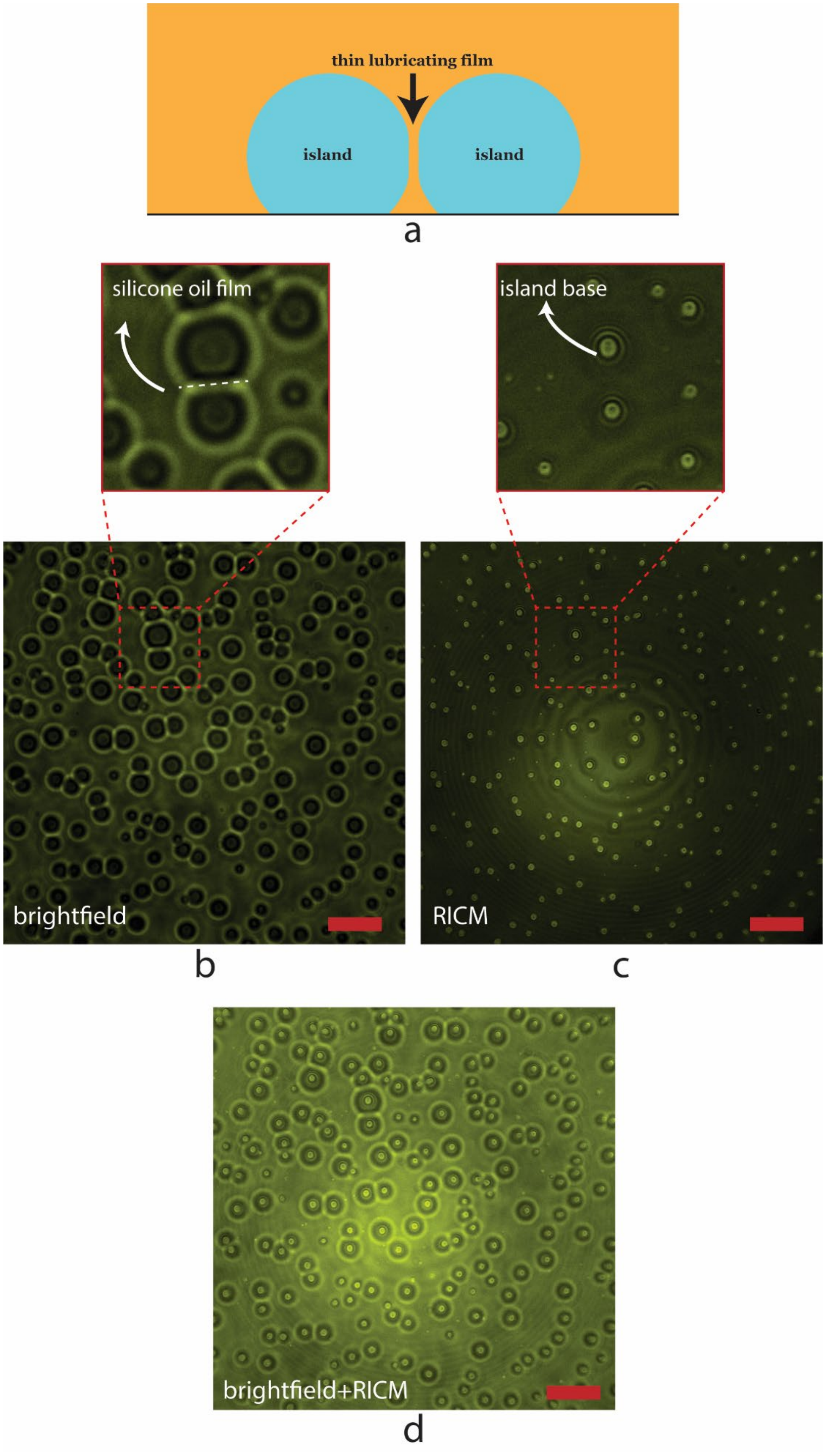

thin lubricating film
island
island
a
silicone oil film
island base
brightfield
RICM
b
c
brightfield+RICM
d

Supplementary Fig. 12. a) Thin film of the suspending film trapped between two islands with contact angles greater than 90° with experimental images showing the same configuration under illumination of b) brightfield, c) reflected mode (RICM) illumination, d) composite image with both brightfield and RICM illumination. Scale bar: 10 μm

It must be noted that oscillatory forces have been recorded in the literature even when small molecule oils such as n-hexane, dodecane, hexadecane, etc. are squeezed down to molecular length scales[33,37–39]. The difference is that interaction forces involved are around three orders of magnitude weaker than those for polymeric melts such as silicone oil[36] and polybutadiene[40]. Consequently, van der Waals forces can cause instability of structured simple oil films as opposed to structured polymeric films relatively easily[38]. This was confirmed by employing small molecule oils such as castor oil or paraffin oil as the suspending medium in our experiments, wherein coalescence between the parent drop and an island was practically instantaneous upon contact. We also checked if smaller molecule impurities such as water can disrupt ordering of castor oil molecules in the form of nanometric films, by conducting experiments with dehydrated castor oil as the suspending phase. The parent drop was glycerol and the substrate chosen was plasma-treated SU8. As seen in Supplementary Fig. 10, similar instantaneous instability is observed for this case, suggesting that the removal of dissolved water in castor oil did not result in a measurable mitigation of the wetting rate.

We further probe as to why oscillatory forces could potentially originate at a liquid/polymer melt interface. de Gennes argued that adsorption and pinning of polymer chains is essential for structuring and layering to occur[35]. Further, various studies have shown the presence of van der Waals and hydrogen bonding between the methyl groups of silicone oil and the hydroxyl groups in silica and mica substrates, leading to an orientation of the polymer chains parallel to the interface[41–45].

Zeng et al. (2009)[46] observed a Stribeck-like friction behavior, commonly observed at solid-solid interfaces, at polybutadiene-silicone oil interfaces. They concluded that there can exist, interdigitated van der Waals bonds between the dissimilar polymer chains, which need to be broken, for the interface to shear past each other. While these experiments were conducted for polymer-polymer interfaces, we explored the literature for observations of attractive forces and structuring at polymer-liquid interfaces. Indeed, such observations have been made at PDMS-water interfaces as well. Molecular dynamics simulations and sum frequency generation spectroscopy studies have shown enhanced structuring and strong hydrogen bonding at PDMS-water interfaces[20,21,47,48]. We expect hydrogen bonding to be even stronger between glycerol and silicone oil molecules due to additional hydroxyl groups in glycerol. In summary, our own experimental observations, coupled with existing literature on the presence of attractive forces between silicone oil chains near an aqueous medium, leading to polymer chain structuring, leads us to believe that the polymer films can be immobilized near liquid-polymer melt interfaces as well.

Supplementary Note 8. Effect of water on polymer confinement

Layering and pinning of silicone polymer chains can be disrupted by the presence of trace amounts of impurities such as dissolved water[32,33,35]. In the experiments that we discuss in this section, we observe that the presence of water facilitates the instantaneous rupture of the silicone oil film when the parent drop meets a glycerol island, as opposed to the situation when the silicone oil is free of impurities, in which case extended growth regimes are attained.

To measure the amount of dissolved water in the silicone oil samples for which 'instantaneous' film rupture occurred, we prepared a set of calibration samples of silicone oil. Small amounts of distilled water were introduced into silicone oil to reach 100% saturation. The excess undissolved water was centrifuged out. To dehydrate silicone oil, we used molecular sieves of pore size 4 Å. The intermediate compositions for the calibration set were obtained by mixing the fully saturated silicone oil (100% saturation) and the completely dehydrated silicone oil sample (0% saturation).

The calibration curve was obtained using a Bruker Hyperion 1000 FTIR, operated in the ATR mode. Peaks were observed at the expected characteristic wavenumber of 3400 cm^{-1} (Supplementary Fig. 13). Using the calibration curves, we were able to determine the saturation level of our silicone oil samples to be about 75%.

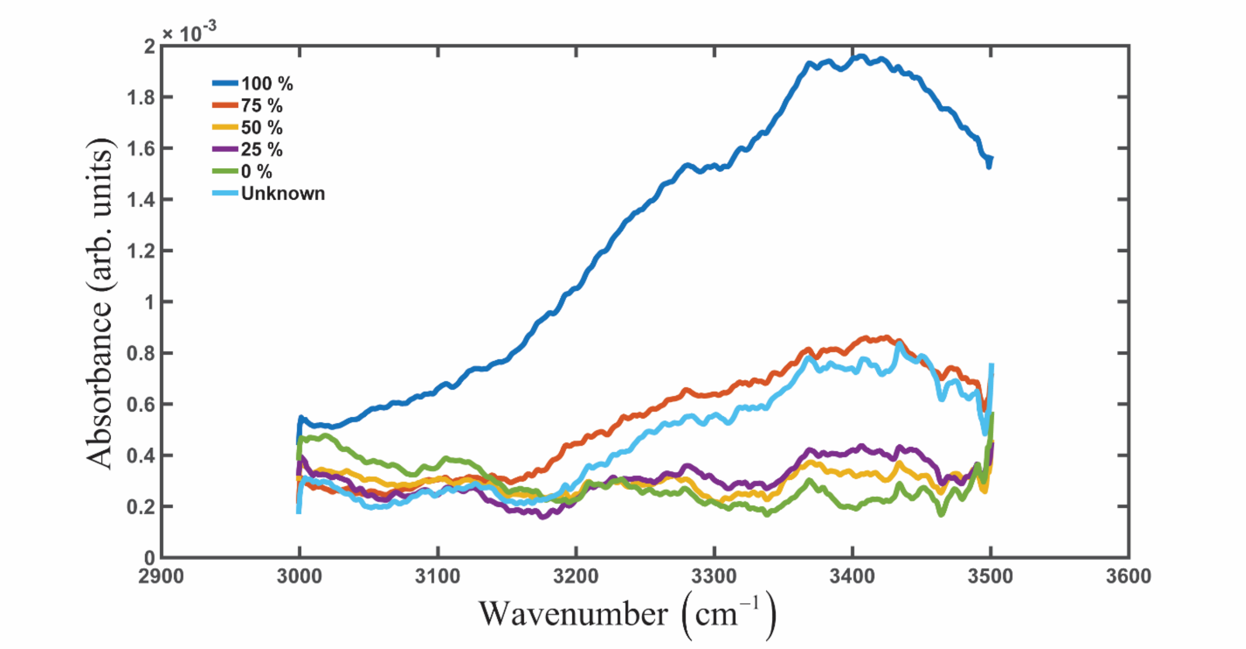

Supplementary Fig. 13. FTIR data for SO1000 with varying levels of moisture for the purpose of calibration. The peak for water occurs at 3400 cm^{-1}. The sample marked 'Unknown' is the sample used in Supplementary Movie 3 (right), and has a saturation in the vicinity of 75% saturation.

Supplementary Movie 7 shows two cases of film drainage between glycerol and plasma-treated SU8 in silicone oil with close to 75% saturation of water and dehydrated silicone oil. While the nucleation and growth of islands is similar in both cases, upon contact of the parent drop with one of the islands, instantaneous film rupture is observed in the case of the silicone with the trace water content.

Supplementary Note 9. Insights from past literature on the stability of nanobubbles and nanodroplets

In this section, we will discuss the various mechanisms proposed in the literature to explain the stability of nanodroplets and nanobubbles, and assess if they can explain the stability of the islands observed in our experiments. We follow the recent detailed review on this topic[49], but adapt it to the situation in this paper.

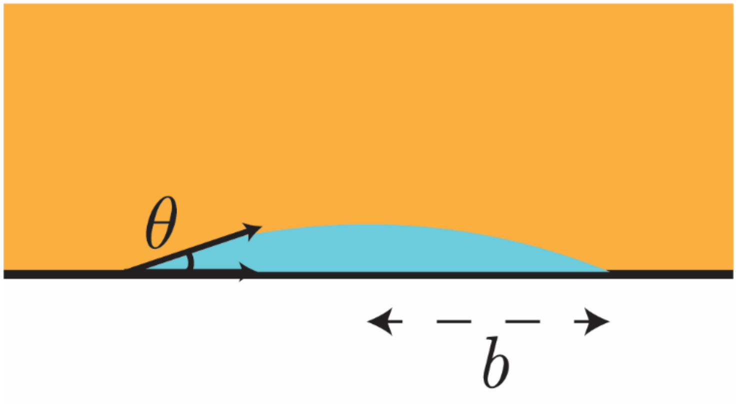

Supplementary Fig. 14. Schematic of an island on a substrate.

Consider an island at the surface of a substrate in the form of a spherical cap of base radius b and contact angle θ as shown in Supplementary Fig. 14. The rate of increase of the island volume with time is governed by mechanisms that add to the volume of the island at the rate $\dot{v}_{IN}$, and mechanisms that deplete the volume of the island at the rate $\dot{v}_{OUT}$. If the contact angle remains constant due to the volume change of the island, then the equation that governs the island radius b is

$$\pi\hat{V}\left(\theta\right)b^2\frac{db}{dt}=\dot{v}_{IN}-\dot{v}_{OUT}. \tag{S9.1}$$

This is equation 2 in the main manuscript. $\hat{V}(\theta)=(1-\cos\theta)^2(2+\cos\theta)/\sin^3\theta$ is a positive function that captures the volume dependence of the island on the contact angle. Let the volume concentration of the dissolved drop fluid on the interface between the island and suspending medium be c_c, and the bulk volume concentration of the dissolved drop fluid be c_∞. Let us assume that $c_c > c_\infty$, which may be anticipated due to the high curvature of the island in our experiments and the negligible curvature of the parent drop. In that case, the volume loss rate from the island due to diffusion, assuming that the ambient medium surrounding the island is saturated with the drop fluid, is given by the equation[50],

$$\dot{v}_{OUT}=\pi Db(c_c-c_\infty)f(\theta), \tag{S9.2}$$

where the function $f(\theta)$ is defined as

$$f(\theta)=\frac{\sin\theta}{1+\sin\theta}+4\int_0^\infty\frac{1+\cosh 2\theta\tau}{\sinh 2\pi\tau}\tanh\left[(\pi-\theta)\tau\right]d\tau \tag{S9.3}$$

and is always positive. Due to the high curvature of the island, we expect

$$c_c\approx c_\infty\left(1+\frac{d\sin\theta}{b}\right). \tag{S9.4}$$

Here, $d=2V_m\sigma/(N_{av}KT)$ is the molecular length scale appearing in the Kelvin equation. If there is no source of volume addition to the island (i.e. $\dot{v}_{IN}=0$), and the drop is far away from the island $(h\gg b)$, then the island base radius b would obey the differential equation,

$$\frac{db}{dt}=-\frac{Dc_\infty\sin\theta f(\theta)}{\hat{V}(\theta)}\frac{1}{b^2}, \tag{S9.5}$$

Thus, if the contact angle remains constant during the volume loss process, the island should shrink monotonically with time due to the diffusive losses, and should eventually vanish. However, this is the opposite of what we see in the experiments; the islands actually *grow* with time.

Several mechanisms have been proposed in the literature as to why the island should be able to grow with time and be stable. These are interfacial mass transfer resistance[51], supersaturation, interfacial impurities[51], surface enhancement and dynamic equilibrium[52–55], and contact line pinning[56]. We will consider these one by one in the sub-sections below.

Interfacial mass transfer resistance

An interfacial mass transfer resistance due to the presence of contaminants and other impurities at the interface that retards the loss of drop fluid to the bulk medium has been proposed in the past as a possibility for explaining the stability of nanobubbles nanodroplets[51]. But as explained previously, it has been largely disfavoured as it falls short upon a quantitative analysis, and is inadequate in explaining stability in experiments where the interfacial mass transfer resistance would be modified or reduced[49]. Besides, the introduction of an interfacial mass transfer resistance only retards the loss rate, and cannot directly lead to addition of volume to the island.

Supersaturation

If the drop fluid is dissolved in the medium at a supersaturated value of $(1+\alpha)c_\infty$, where the parameter α is positive, then

$$\frac{db}{dt}=\frac{Dc_\infty f(\theta)}{\hat{V}(\theta)}\frac{1}{b}\left(\alpha-\frac{d\sin\theta}{b}\right), \tag{S9.6}$$

The supersaturation in the suspending medium in our experiments could arise due to the presence of the parent drop, which has low but finite curvature. Thus, $\xi \approx d/R$, where R is the radius of the drop, and hence

$$\frac{db}{dt} = \frac{Dc_\infty f(\theta)}{\hat{V}(\theta)} \frac{d}{b}\left(\frac{1}{R} - \frac{\sin\theta}{b}\right), \tag{S9.7}$$

The above equation suggests a steady island radius, but it is unstable. If the initial island radius is below $R\sin\theta$, the island will dissolve in the suspending medium and disappear completely. But if the island radius is above $R\sin\theta$, then the island will grow continuously. In our experiments, the smallest drop radii are about 30 μm, and the smallest contact angle is about 20°, implying that $R\sin\theta$ is at least about 10 μm. This means that an island of radius greater than 10 μm would have to be nucleated for it to grow continuously. In our experiments, we can clearly see islands that are about a micron in diameter. We can also start to see traces of growing islands in the RICM images even when it is sub-micron in size. Hence, supersaturation cannot explain the observed growth of the islands.

<u>Dynamic equilibrium theory</u>

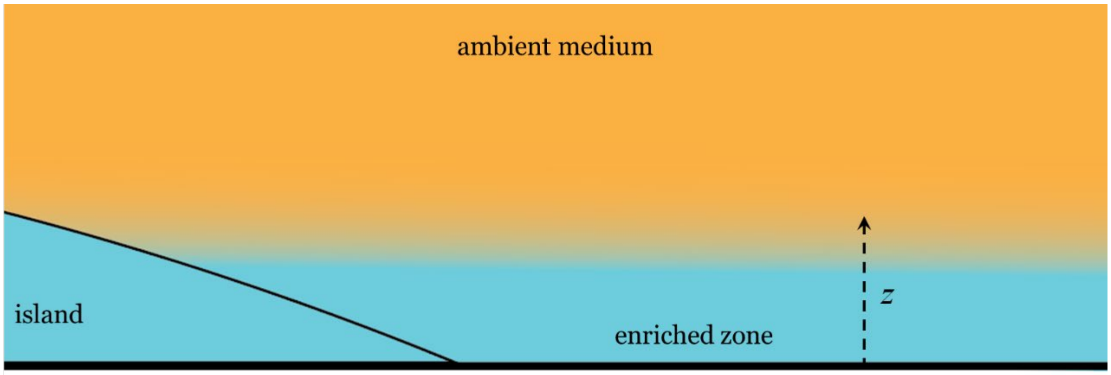

Supplementary Fig. 15. Schematic of a zone near the substrate enriched with dissolved phase due to an attractive potential ϕ.

According to the dynamic equilibrium theory, an attractive potential ϕ between the dissolved drop fluid molecules and the substrate sets up a thin zone in the vicinity of the surface that is enriched in the dissolved drop fluid. If z is the co-ordinate normal to the substrate, then the concentration distribution is set up according to the equilibrium equation,

$$-D\frac{dc}{dz}-c\zeta\frac{d\phi}{dz}=0, \tag{S9.8}$$

where ζ is the solute mobility, equal to D/KT. Here K is the Boltzmann constant, and T is the absolute temperature. An island growing on the substrate intercepts this region of higher drop fluid concentration along the contact line as shown in Supplementary Fig. 15., and the concentration gradient normal to the island-medium interface in the enriched zone provides a source of influx of drop fluid molecules to the island. In the limit that the zone of enrichment is much thinner than the height of the island, the rate of volume addition to the island takes the form,

$$\dot{v}_{IN}\approx 2\pi bD\beta c_c\sin\theta\cos\theta. \tag{S9.9}$$

There are two problems with the dynamic equilibrium theory. First, as has been pointed out by different authors, the ultimate driving force for the exchange of mass between the island and the bulk liquid is not understood[49,52,53]. Second, it attributes mass transfer to gradients in solute concentration. But it is well known that mass transfer results from gradients in chemical potential, not concentration[57]. Even though the region near the substrate may be enriched in drop fluid due to an attractive potential, the gradient in chemical potential is zero if the surface has reached equilibrium with the bulk. To see this, consider the following simplified version of the equation S9.8,

$$-\zeta c\frac{dM}{dz}=0. \tag{S9.10}$$

Here, $M = KT \ln c + \phi$ is a chemical potential that includes the attractive potential function ϕ.

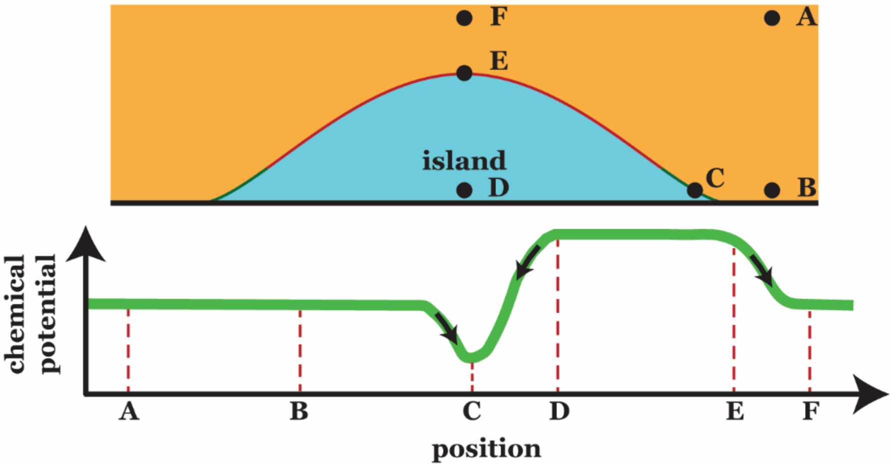

Supplementary Fig. 16. Chemical potential variation at various locations around an island on a substrate.

The above equation suggests that the chemical potential is a constant everywhere in the bulk. So if an island is placed on the substrate, there should be no difference in terms of mass transfer between a drop fluid molecule in the bulk (point A in Supplementary Fig. 16), and a drop fluid molecule near the substrate (point B in Supplementary Fig. 16). Since the chemical potential on the island surface (point E in Supplementary Fig. 16) is greater than the chemical potential at point A (which leads to Ostwald ripening effects), it would be greater than the chemical potential at point B. This implies that the drop would *lose* volume to the enriched zone near the surface, and not receive drop molecules from it.

<u>**Drop pinning effects**</u>

In the presence of a supersaturation, if the contact line is pinned, the island radius b remains constant, and contact angle adjusts with time to accommodate island volume changes. The differential equation governing the contact angle is

$$\frac{d\theta}{dt} = \frac{3Dc_{\infty}}{b^2} \frac{f(\theta)}{\hat{V}'(\theta)} \left(\alpha - \frac{d \sin\theta}{b} \right), \tag{S9.11}$$

which allows a steady state contact angle governed by

$$\sin\theta = \frac{\alpha b}{d}. \tag{S9.12}$$

A linear stability analysis shows that the above steady state is stable. A positive perturbation in volume about this steady state leads to an increase in the contact angle at a fixed base radius, and hence a decrease in the radius of curvature, which increases the island solubility and hence the loss rate to return the volume back to the steady state. A similar argument would explain the stability with respect to negative perturbations in the island volume about the steady state. Unfortunately, we do not expect to see such a steady state in our experiments, because the contact lines of the islands are not pinned.

Shielding effect

The ability of neighbouring islands to reduce the ambient concentration gradient has been previously shown in literature[58–65]. It has been argued that this can extend the lifetimes of dissolving nanobubbles. Similar to our previous arguments, while this mechanism can cause the islands to dissolve slowly, it does not explain why the islands would grow as a function of time. Moreover, in our experiments, we have observed instances where growing islands are far apart that the influence of neighbouring islands would be minimal.

The inability of existing mechanisms in the literature to explain the continuous growth of islands in our experiments led us to explore the alternative mechanism based on the reduction of solubility by an attractive disjoining pressure, as discussed in the main manuscript.

Supplementary Note 10. Constancy of contact angle during island growth

The long time experiments wherein the glycerol islands are allowed to grow below a larger parent glycerol drop, confirm that the islands maintain a constant contact angle during growth. This result is important for the estimation of the critical height of rupture as shown in Supplementary note 15. If we assume the glycerol islands in our experiments to be spherical caps (a reasonable approximation for the island sizes under consideration), the island height ℓ, the base radius b and the contact angle θ, are related by $\ell = b\tan(\theta/2)$. The island height can be separately measured by counting the interference fringes and the base radius can be measured using the outer edge of the islands. A plot of ℓ vs b would then yield a straight line passing through the origin (0,0), provided the contact angle is constant; this is indeed what is observed (see Supplementary Fig. 17).

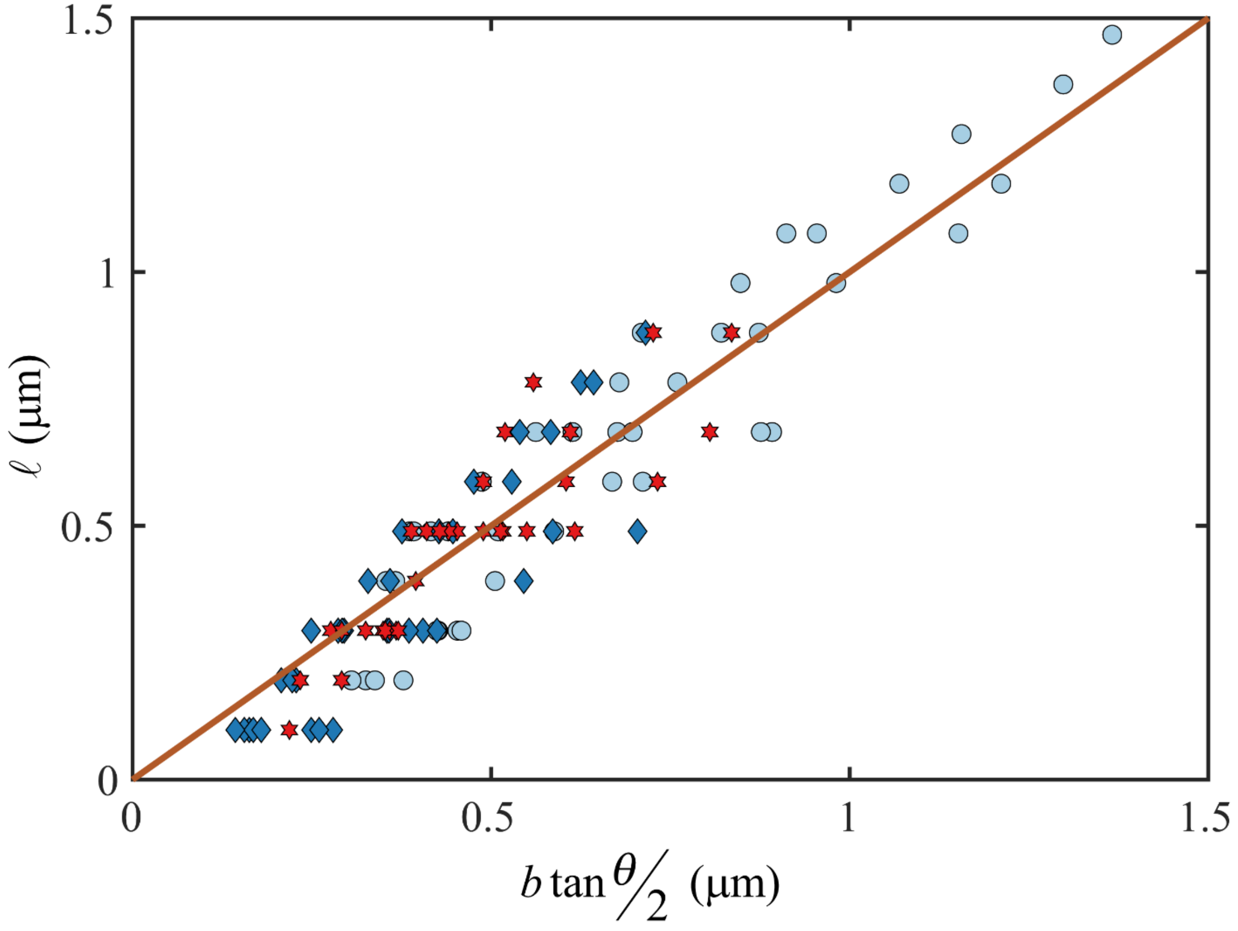

Supplementary Fig. 17. A plot of island height ℓ against $b\tan\theta/2$, where b is the base radius and θ is the contact angle. The data is shown for the SO500-G-M (♦), SO1000-G-M (●) and SO1000-G-PS (✶) systems. Since ℓ is proportional to b for each case, the contact angle is a constant during the growth of the island. The parity plot of ℓ against $b\tan\theta/2$ shown above was generated by deducing the contact angles from the slope of the ℓ vs b plots. The inferred contact angles of glycerol islands are $15.8^{\circ}\pm1.3^{\circ}$, $16.2^{\circ}\pm0.5^{\circ}$ and $31.7^{\circ}\pm2.1^{\circ}$ for the SO500-G-M, SO1000-G-M and SO1000-G-PS systems, respectively.

Supplementary Note 11. Lower bound for length scale over which disjoining pressure is prevalent

The explanations of island growth in the discussions of island stability are founded on the inherent assumption that $\dot{v}_{OUT} << \dot{v}_{IN}$, which assures the stability of the islands. It is instructive to examine the model to elucidate the regime of parameters for which this assumption is true.

<u>Argument for $b^3 \sim t$</u>

When the drop is far away from the island, $\dot{v}_{OUT} \sim Db\left(c_c - c_\infty\right)$ and $\dot{v}_{IN} \sim Dz_0 c_\infty / \sin\theta$, and hence, $\dot{v}_{OUT} \ll \dot{v}_{IN}$ requires that $\left[\left(c_c - c_\infty\right)/c_\infty\right] \ll z_0 / \left(b\sin\theta\right)$. Since $z_0 / b << 1$ for most cases of island sizes, the dimensionless concentration difference $\left(c_c - c_\infty\right)/c_\infty$ has to be weak for the islands to be stable, i.e. the medium has to be close to saturation $c_c \approx c_\infty$. Thus, island growth should certainly not be observed when the medium is devoid of the drop fluid $\left(c_\infty = 0\right)$. When the island is small, $\left(c_c - c_\infty\right)/c_\infty \approx d\sin\theta / b$, where $d = 2V_m\sigma / \left(N_a KT\right)$ is the molecular length scale appearing in the Kelvin equation. Hence, for small islands to be stable, $z_0 \gg d\sin^2\theta$. For glycerol in silicone oil, the molar volume $V_m = 73.1$ cm³/mol, $\sigma = 30$ mN/m, $R = 8.314$ J/mol-K and $T = 298$ K, d can be calculated to be 1.8 nm. For contact angles θ ranging from 30° to 50° (the range of contact angles observed in our work when plasma treated SU-8 was the substrate), the length scale z_0 of influence of the disjoining pressure on the solubility must exceed 0.45-1.1 nm, or about 1 nm.

Argument for $b^2 \sim t$

When the drop is far away from the island, $\dot{v}_{OUT} \sim Db\left(c_c - c_\infty\right)$ and $\dot{v}_{IN} \sim \dfrac{D_{im} z_0 b c_\infty}{d_{im} \sin\theta}$, and hence, $\dot{v}_{OUT} \ll \dot{v}_{IN}$ requires that $\left[\left(c_c - c_\infty\right)/c_\infty\right] \ll \left(D_{im}/D\right) z_0 / \left(d_{im} \sin\theta\right)$. Since $\left(D_{im}/D\right) << 1$, the dimensionless concentration difference $\left(c_c - c_\infty\right)/c_\infty$ has to be weak for the islands to be stable, i.e. the medium has to be close to saturation $c_c \approx c_\infty$. As in the previous case, island growth should not be observed when the medium is devoid of the drop fluid $\left(c_\infty = 0\right)$. From Kelvin's equation, $\left(c_c - c_\infty\right)/c_\infty \approx d \sin\theta / b$, where $d = 2V_m \sigma / \left(RT\right)$ is the molecular length scale appearing in the Kelvin equation. Hence, for small islands to be stable, $z_0 \gg d_{im} d \sin^2\theta \left(D/D_{im}\right)/b$. Taking the lower limit for z_0, we get the expression $z_0 = d_{im} d \sin^2\theta \left(D/D_{im}\right)/b$, which is a function of the island base radius b. Substituting the respective parameter values for glycerol in silicone oil: $d = 1.8$ nm, $d_{im} = 10$ nm, $D = 10^{-11}$ $\mathrm{m^2/s}$, $D_{im} = 10^{-15}$ $\mathrm{m^2/s}$ and $\theta = 17^\circ$, a plot of z_0 vs b shows unrealistically large values of z_0 for small b (see Supplementary Fig. 18, red curve). This is not surprising because the above analysis assumes that influx takes place at the contact line which is always submerged in the immobilized polymer layer, while the outflux takes place across the curved surface area of the island exposed to the bulk polymer melt. The diffusivity of glycerol being 4 orders of magnitude smaller in the immobilized layer, then requires unrealistically long-ranged $\left(z_0 \sim 10\ \mu\mathrm{m}\right)$ attractive forces to exist for the islands to be stable. A plot of island height ℓ vs b, however, shows that until a base radius of about 100 nm, the island is completely submerged inside the immobilized layer. Hence, for base radii less than 100 nm, the appropriate diffusion coefficient for outflux is not D, but D_{im}, which leads to overestimation of the loss rate. The correct minimum value of z_0 for these base

radii is given by $z_0 = d_{im} d \sin^2\theta / b$, yielding values of z_0 to be about 1 nm (Supplementary Fig. 18, green curve), which are more consistent with the growth of the island. Further, for base radii greater than about 1 μm, reasonable values of $z_0 = 10$ nm and below, can be observed as shown in Supplementary Fig. 18. We believe that island growth cannot be sustained for island radii between 100 nm- 1 μm unless coalescence of several adjacent islands occur. This could also explain the observation that in the SO1000-G-M and SO500-G-M cases, islands are relatively sparse and more difficult to observe as compared to SO1000-G-PS.

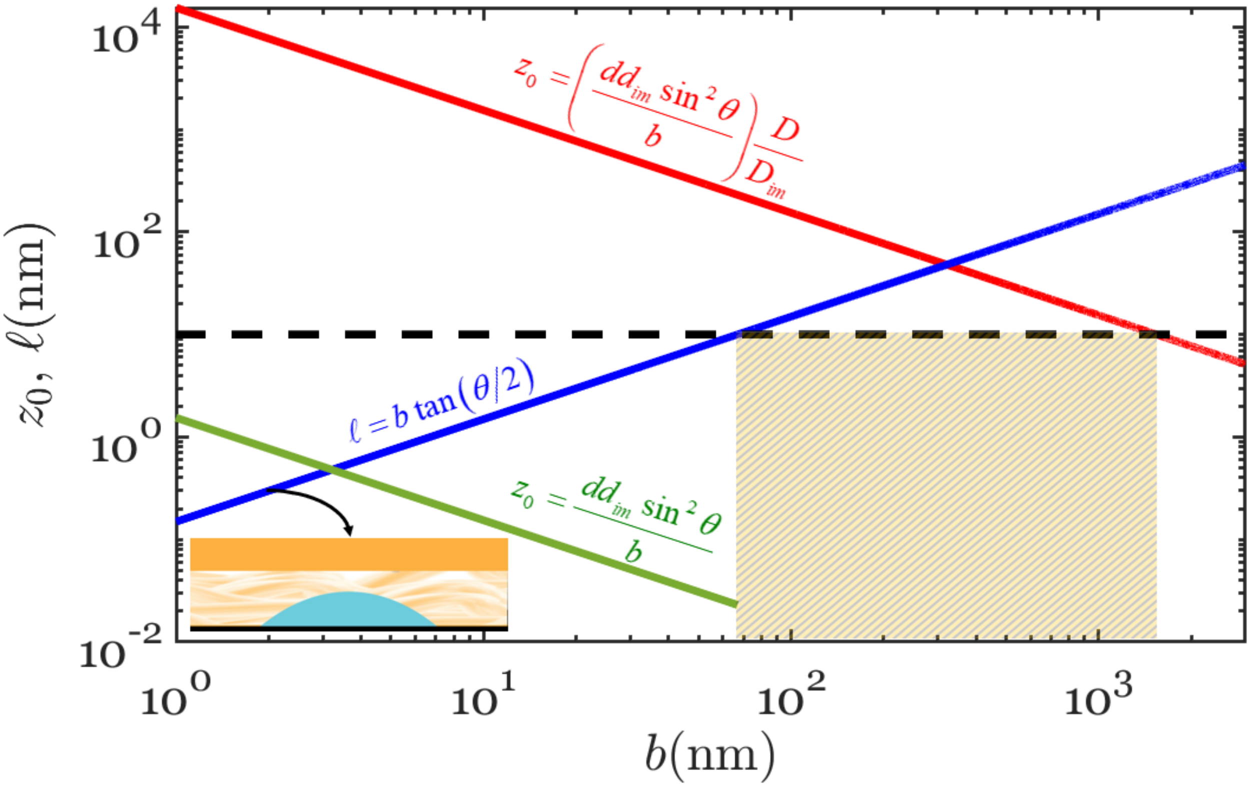

Supplementary Fig. 18. Plot of lower bound of length scale (z_0) over which disjoining pressure acts, as a function of island base radius b. The red curve denotes the case where major portions of the island is above the immobilized polymer layer. The green curve is the scenario where the island is completely submerged in the immobilized region. The blue curve is the island height (ℓ) as a function of base radius to show when the island emerges out of the immobilized layer. The yellow hashed region for island base radii 100 nm-1 μm, is a region of ambiguity where island growth can occur only by coalescence of neighbouring islands

until the island radius reaches about 1 μm. The plot was constructed using the following parameter values: $d = 1.8$ nm, $d_{im} = 10$ nm, $D = 10^{-11}$ m^2/s, $D_{im} = 10^{-15}$ m^2/s, $\theta = 17^\circ$.

Supplementary Note 12. Solubility measurement using Nuclear Magnetic Resonance (NMR) spectroscopy

To determine the solubility of glycerol in silicone oil, quantitative measurements of NMR spectra were conducted on the pre-saturated silicone oil (Supplementary Fig. 19). The samples were dissolved in deuterated chloroform (CDCl3). Proton spectra of the samples were conducted at the same conditions: relaxation delay 25 sec and 128 scans. Data were collected on an Agilent DD2 700 MHx spectrometer equipped with H-19F {13C/15N}, 5 mm Triple Resonance Cold Probe. The solubility for both samples, SO500 and SO1000, was found to be 2% by volume.

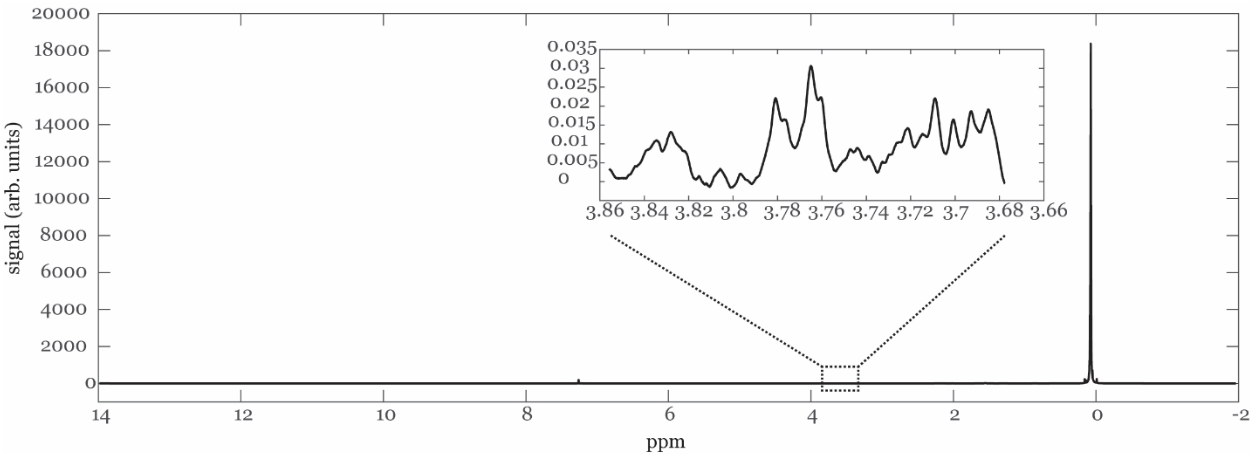

Supplementary Fig. 19. Proton NMR spectrum of silicone oil (1000 cP) saturated with glycerol. The inset shows signals corresponding to glycerol.

Supplementary Note 13. Solubility measurement using confocal Raman spectroscopy

In the previous section (Supplementary Note 12), solubility was measured by presaturation of silicone oil with glycerol by emulsification followed by centrifugation, to remove undissolved droplets of glycerol. However, the accessible centrifugal forces were enough only to remove glycerol droplets that were greater than 1 μm. There was, thus, a possibility that the solubility measured using NMR spectroscopy (Supplementary Note12), was an overestimation due to trace glycerol droplets. In order to validate the measurement, we measured the solubility using confocal Raman spectroscopy using an experimental configuration that does not require emulsification of glycerol in silicone oil.

Raman spectra were obtained using a Renishaw inVia confocal Raman spectrometer and a Leica DMI600 epifluorescence microscope. A 100X (N.A. 1.3) oil immersion objective lens with a depth resolution of about 2 μm, was used to focus a 100 mW, 532 nm laser on to the sample. The acquisition time was set to be 60 s. The scattered Raman light was then dispersed using a 1800 l/mm grating and detected using a 1024 x 256 deep depletion RenCam CCD camera.

To measure the solubility of glycerol in silicone oil, the experimental protocol was as follows: A silicone oil droplet (drop volume ~1 nL) was introduced onto a No.0 glass coverslip in air (Supplementary Fig. 20 a). The silicone oil droplet spreads on the coverslip with a contact angle of about 2°. The air was then replaced with glycerol. The silicone oil droplet now slowly retracts to adjust to a new contact angle of 123°. During this process, glycerol already diffuses into and saturates the silicone oil droplet (For a diffusivity of $D \sim 10^{-11}$ m^2/s, to diffuse over a length scale of 1 μm, takes 0.1 s). Raman spectra was then obtained at two locations: 1) in the glycerol phase (location 1 in Supplementary Fig. 20 a), 2) near the center of the silicone oil droplet. The corresponding Raman spectra are shown in Supplementary Fig. 20 b. After baseline subtraction, the area under the curve for the Raman band between 1006 cm^{-1} and 1160 cm^{-1}. The ratio of the area for location 2 (silicone oil drop) to that at location 1 (glycerol phase) gives the concentration

(volume%) of dissolved glycerol in the silicone oil droplet. The solubility was thus measured to be 1.6 volume%.

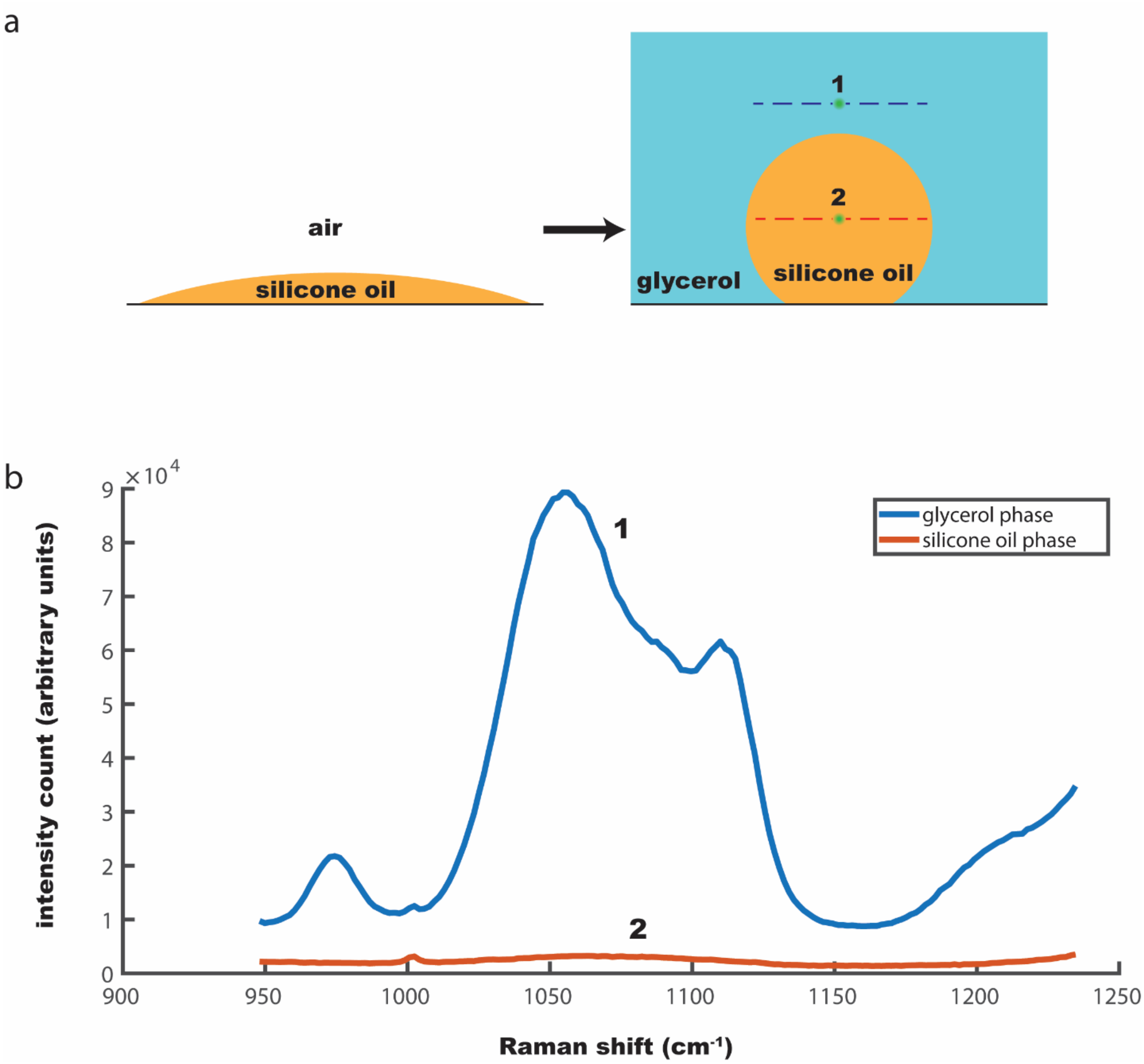

Supplementary Fig. 20. a) Schematic of experimental configuration for confocal Raman spectroscopy. Raman spectra are obtained at locations 1 and 2 after replacing air with glycerol. b) Raman spectra at locations 1 and 2 as depicted in a).

Supplementary Note 14. Estimation of viscosity of immobilized silicone oil layer

The viscosity of the immobilized layer of silicone oil on mica was estimated by observing coalescence events of glycerol islands. When two emulsion drops coalesce to form a larger drop, the relaxation of the drop shape towards a spherical shape is governed by a capillary time scale, given by

$$t \sim \frac{\mu R}{\sigma} \quad \text{(S14.1)}$$

where μ is the viscosity of either the drop or the suspending medium, whichever being higher. Based on Supplementary Movie 10, we can see that it takes 10 mins for an island of radius 5 μm to relax to a spherical shape. Using equation S14.1, and assuming that there is no contact line pinning for a smooth, freshly cleaved mica surface, we can calculate the viscosity of the immobilized layer to be $\mu \sim O\left(10^6\,\mathrm{Pa\ s}\right)$.

Supplementary Note 15. Concentration boundary layer near the drop interface

In this section, we estimate the time required for the drop to wet the surface by merging with an island, assuming that the growth of the island is the rate determining step in the wetting process. To determine this time, we first need to know the film height h_0 at which the surface first 'sees' the glycerol presented by the drop, since this event will trigger the nucleation and growth of the glycerol islands. As the drop descends towards the surface, a boundary layer of dissolved glycerol is present around the settling glycerol drop (see Fig. 5a,b). The thickness, δ, of this concentration boundary layer can be estimated by performing a balance between the time scale of diffusion of dissolved glycerol normal to the drop surface, and the time scale corresponding to convection of glycerol past the drop surface[12].

$$\frac{\delta^2}{D} \sim \frac{a}{U}. \tag{S15.1}$$

Here, D is the diffusivity of glycerol in the suspending medium, a is the film radius and U is the interfacial velocity. The interfacial velocity, U, should scale as the parabolic component of velocity, Gh^2/μ. The pressure gradient, G, can be written as the

$$G \sim \frac{\left(\text{Force}/\text{Area}\right)}{\text{Length scale}} \sim \frac{\left(\Delta\rho g R^3/a\right)}{l_c}. \tag{S15.2}$$

For a spherical film, the length scale, l_c, is equal to the radial length scale, $a = \sqrt{Rh}$. Plugging this into equation (S15.1), we get the thickness of the boundary layer as

$$\delta \sim \sqrt{\frac{\mu D}{\Delta\rho g}} R^{-1/2}. \tag{S15.3}$$

When the boundary layer thickness is of the order of the film thickness, i.e. $h \sim \delta$, the surface begins to receive dissolved glycerol.

The drop meets the island when the film thickness reaches a critical value, h_c, equal to the height of the island, ℓ. Mathematically, h_c can be estimated by simultaneously solving the equation for $b(t)$ and geometrically relating $\ell = b\tan(\theta/2)$ with the equation for film drainage (see Supplementary Table 1 in Supplementary Note 2). The schematics for prediction of the critical height h_c is shown in Supplementary Fig. 21.

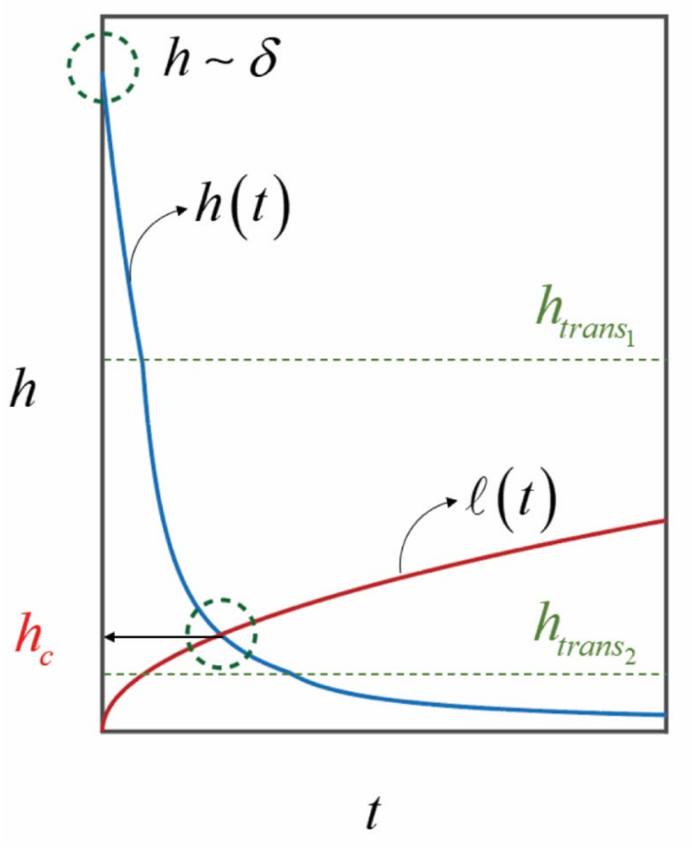

Supplementary Fig. 21. Prediction of critical height h_c with the knowledge of film drainage dynamics $(h(t))$, island growth dynamics $(\ell(t))$ and the boundary layer thickness (δ).

Supplementary References

1. Wiegand, G., Neumaier, K. R. & Sackmann, E. Microinterferometry: Three-Dimensional Reconstruction of Surface Microtopography for Thin-Film and Wetting Studies by Reflection Interference Contrast Microscopy (RICM). *Appl. Opt.* **37**, 6892–6905 (1998).
2. Contreras-Naranjo, J. C., Silas, J. A. & Ugaz, V. M. Reflection interference contrast microscopy of arbitrary convex surfaces. *Appl. Opt.* **49**, 3701–3712 (2010).
3. Contreras-Naranjo, J. C. & Ugaz, V. M. A nanometre-scale resolution interference-based probe of interfacial phenomena between microscopic objects and surfaces. *Nat. Commun.* **4**, ncomms2865 (2013).
4. Limozin, L. & Sengupta, K. Quantitative reflection interference contrast microscopy (RICM) in soft matter and cell adhesion. *Chemphyschem Eur. J. Chem. Phys. Phys. Chem.* **10**, 2752–2768 (2009).
5. Daniel, D., Timonen, J. V. I., Li, R., Velling, S. J. & Aizenberg, J. Oleoplaning droplets on lubricated surfaces. *Nat. Phys.* **13**, 1020–1025 (2017).
6. Sternberg. Biomedical Image Processing. *Computer* **16**, 22–34 (1983).
7. Illingworth, J. & Kittler, J. The Adaptive Hough Transform. *IEEE Trans. Pattern Anal. Mach. Intell.* **PAMI-9**, 690–698 (1987).
8. Bartels, R. H., Beatty, J. C. & Barsky, B. A. *An Introduction to Splines for Use in Computer Graphics & Geometric Modeling*. (Morgan Kaufmann Publishers Inc., 1987).
9. Yiantsios, S. G. & Davis, R. H. On the buoyancy-driven motion of a drop towards a rigid surface or a deformable interface. *J. Fluid Mech.* **217**, 547–573 (1990).
10. Ramachandran, A. & Leal, L. G. Effect of interfacial slip on the thin film drainage time for two equal-sized, surfactant-free drops undergoing a head-on collision: A scaling analysis. *Phys. Rev. Fluids* **1**, 064204 (2016).

11. Frostad, J. M., Collins, M. C. & Leal, L. G. Cantilevered-Capillary Force Apparatus for Measuring Multiphase Fluid Interactions. *Langmuir* **29**, 4715–4725 (2013).

12. Leal, L. G. *Advanced Transport Phenomena: Fluid Mechanics and Convective Transport Processes*. (Cambridge University Press, 2007).

13. Goel, S. & Ramachandran, A. The suppression of droplet-droplet coalescence in a sheared yield stress fluid. *J. Colloid Interface Sci.* **492**, 199–206 (2017).

14. Chhabra, R. P., Uhlherr, P. H. T. & Boger, D. V. The influence of fluid elasticity on the drag coefficient for creeping flow around a sphere. *J. Non-Newton. Fluid Mech.* **6**, 187–199 (1980).

15. Rykaczewski, K., Landin, T., Walker, M. L., Scott, J. H. J. & Varanasi, K. K. Direct Imaging of Complex Nano- to Microscale Interfaces Involving Solid, Liquid, and Gas Phases. *ACS Nano* **6**, 9326–9334 (2012).

16. Anand, S., Rykaczewski, K., Subramanyam, S. B., Beysens, D. & Varanasi, K. K. How droplets nucleate and grow on liquids and liquid impregnated surfaces. *Soft Matter* **11**, 69–80 (2014).

17. Cheng, N.-S. Formula for the Viscosity of a Glycerol−Water Mixture. *Ind. Eng. Chem. Res.* **47**, 3285–3288 (2008).

18. Takamura, K., Fischer, H. & Morrow, N. R. Physical properties of aqueous glycerol solutions. *J. Pet. Sci. Eng.* **98–99**, 50–60 (2012).

19. Walther, F. *et al.* Surface hydrophilization of SU-8 by plasma and wet chemical processes. *Surf. Interface Anal.* **42**, 1735–1744 (2010).

20. Ismail, A. E., Grest, G. S., Heine, D. R., Stevens, M. J. & Tsige, M. Interfacial Structure and Dynamics of Siloxane Systems: PDMS−Vapor and PDMS−Water. *Macromolecules* **42**, 3186–3194 (2009).

21. Tian, C. S. & Shen, Y. R. Structure and charging of hydrophobic material/water interfaces studied by phase-sensitive sum-frequency vibrational spectroscopy. *Proc. Natl. Acad. Sci.* **106**, 15148–15153 (2009).

22. Vácha, R., Zangi, R., Engberts, J. B. F. N. & Jungwirth, P. Water Structuring and Hydroxide Ion Binding at the Interface between Water and Hydrophobic Walls of Varying Rigidity and van der Waals Interactions. *J. Phys. Chem. C* **112**, 7689–7692 (2008).

23. Björneholm, O. *et al.* Water at Interfaces. *Chem. Rev.* **116**, 7698–7726 (2016).

24. Agmon, N. *et al.* Protons and Hydroxide Ions in Aqueous Systems. *Chem. Rev.* **116**, 7642–7672 (2016).

25. Yaminsky, V. V., Ohnishi, S., Vogler, E. A. & Horn, R. G. Stability of Aqueous Films between Bubbles. Part 1. The Effect of Speed on Bubble Coalescence in Purified Water and Simple Electrolyte Solutions. *Langmuir* **26**, 8061–8074 (2010).

26. Ivanov, I. B. & Kralchevsky, P. A. Stability of emulsions under equilibrium and dynamic conditions. *Colloids Surf. Physicochem. Eng. Asp.* **128**, 155–175 (1997).

27. Karraker, K. A. & Radke, C. J. Disjoining pressures, zeta potentials and surface tensions of aqueous non-ionic surfactant/electrolyte solutions: theory and comparison to experiment. *Adv. Colloid Interface Sci.* **96**, 231–264 (2002).

28. Leunissen, M. E., Blaaderen, A. van, Hollingsworth, A. D., Sullivan, M. T. & Chaikin, P. M. Electrostatics at the oil–water interface, stability, and order in emulsions and colloids. *Proc. Natl. Acad. Sci.* **104**, 2585–2590 (2007).

29. Creux, P., Lachaise, J., Graciaa, A., Beattie, J. K. & Djerdjev, A. M. Strong Specific Hydroxide Ion Binding at the Pristine Oil/Water and Air/Water Interfaces. *J. Phys. Chem. B* **113**, 14146–14150 (2009).

30. Gu, Y. & Li, D. The ζ-Potential of Silicone Oil Droplets Dispersed in Aqueous Solutions. *J. Colloid Interface Sci.* **206**, 346–349 (1998).

31. Serjeant, E. P. & Dempsey, B. *Ionisation constants of organic acids in aqueous solution.* (Pergamon Press, 1979).

32. Klein, J. & Kumacheva, E. Simple liquids confined to molecularly thin layers. I. Confinement-induced liquid-to-solid phase transitions. *J. Chem. Phys.* **108**, 6996–7009 (1998).

33. Kumacheva, E. & Klein, J. Simple liquids confined to molecularly thin layers. II. Shear and frictional behavior of solidified films. *J. Chem. Phys.* **108**, 7010–7022 (1998).

34. Evmenenko, G., Dugan, S. W., Kmetko, J. & Dutta, P. Molecular Ordering in Thin Liquid Films of Polydimethylsiloxanes. *Langmuir* **17**, 4021–4024 (2001).

35. Horn, R. G. & Israelachvili, J. N. Molecular organization and viscosity of a thin film of molten polymer between two surfaces as probed by force measurements. *Macromolecules* **21**, 2836–2841 (1988).

36. Israelachvili, J. N. & Kott, S. J. Liquid structuring at solid interfaces as probed by direct force measurements: The transition from simple to complex liquids and polymer fluids. *J. Chem. Phys.* **88**, 7162–7166 (1988).

37. Christenson, H. K., Gruen, D. W. R., Horn, R. G. & Israelachvili, J. N. Structuring in liquid alkanes between solid surfaces: Force measurements and mean-field theory. *J. Chem. Phys.* **87**, 1834–1841 (1987).

38. Gee, M. L., McGuiggan, P. M., Israelachvili, J. N. & Homola, A. M. Liquid to solidlike transitions of molecularly thin films under shear. *J. Chem. Phys.* **93**, 1895–1906 (1990).

39. Klein, J. & Kumacheva, E. Confinement-Induced Phase Transitions in Simple Liquids. *Science* **269**, 816–819 (1995).

40. Luengo, G., Schmitt, F.-J., Hill, R. & Israelachvili, J. Thin Film Rheology and Tribology of Confined Polymer Melts: Contrasts with Bulk Properties. *Macromolecules* **30**, 2482–2494 (1997).

41. Smith, J. S., Borodin, O., Smith, G. D. & Kober, E. M. A molecular dynamics simulation and quantum chemistry study of poly(dimethylsiloxane)–silica nanoparticle interactions. *J. Polym. Sci. Part B Polym. Phys.* **45**, 1599–1615 (2007).

42. Tsige, M. *et al.* Interactions and structure of poly(dimethylsiloxane) at silicon dioxide surfaces: Electronic structure and molecular dynamics studies. *J. Chem. Phys.* **118**, 5132–5142 (2003).

43. Evmenenko, G., Mo, H., Kewalramani, S. & Dutta, P. X-ray Reflectivity Study of Ultrathin Liquid Films of Diphenylsiloxane−Dimethylsiloxane Copolymers. *Langmuir* **22**, 6245–6248 (2006).

44. Bae, S. C., Lee, H., Lin, Z. & Granick, S. Chemical Imaging in a Surface Forces Apparatus: Confocal Raman Spectroscopy of Confined Poly(dimethylsiloxane). *Langmuir* **21**, 5685–5688 (2005).

45. Jiang, S., Bae, S. C. & Granick, S. PDMS Melts on Mica Studied by Confocal Raman Scattering. *Langmuir* **24**, 1489–1494 (2008).

46. Zeng, H., Tian, Y., Zhao, B., Tirrell, M. & Israelachvili, J. Friction at the Liquid/Liquid Interface of Two Immiscible Polymer Films. *Langmuir* **25**, 4954–4964 (2009).

47. Tian, C. S. & Shen, Y. R. Recent progress on sum-frequency spectroscopy. *Surf. Sci. Rep.* **69**, 105–131 (2014).

48. Strazdaite, S., Versluis, J., Backus, E. H. G. & Bakker, H. J. Enhanced ordering of water at hydrophobic surfaces. *J. Chem. Phys.* **140**, 054711 (2014).

49. Lohse, D. & Zhang, X. Surface nanobubbles and nanodroplets. *Rev. Mod. Phys.* **87**, 981–1035 (2015).

50. Popov, Y. O. Evaporative deposition patterns: Spatial dimensions of the deposit. *Phys. Rev. E* **71**, 036313 (2005).

51. Ducker, W. A. Contact Angle and Stability of Interfacial Nanobubbles. *Langmuir* **25**, 8907–8910 (2009).

52. Brenner, M. P. & Lohse, D. Dynamic Equilibrium Mechanism for Surface Nanobubble Stabilization. *Phys. Rev. Lett.* **101**, 214505 (2008).

53. Petsev, N. D., Shell, M. S. & Leal, L. G. Dynamic equilibrium explanation for nanobubbles' unusual temperature and saturation dependence. *Phys. Rev. E* **88**, 010402 (2013).

54. Tan, B. H., An, H. & Ohl, C.-D. Surface Nanobubbles Are Stabilized by Hydrophobic Attraction. *Phys. Rev. Lett.* **120**, 164502 (2018).

55. Tan, B. H., An, H. & Ohl, C.-D. Stability, Dynamics, and Tolerance to Undersaturation of Surface Nanobubbles. *Phys. Rev. Lett.* **122**, 134502 (2019).

56. Weijs, J. H. & Lohse, D. Why surface nanobubbles live for hours. *Phys. Rev. Lett.* **110**, 054501 (2013).

57. Deen, W. M. *Analysis of Transport Phenomena*. (Oxford University Press, 2012).

58. Laghezza, G. *et al.* Collective and convective effects compete in patterns of dissolving surface droplets. *Soft Matter* **12**, 5787–5796 (2016).

59. Bao, L. *et al.* Flow-induced dissolution of femtoliter surface droplet arrays. *Lab. Chip* **18**, 1066–1074 (2018).

60. Schofield, F. G. H., Wray, A. W., Pritchard, D. & Wilson, S. K. The shielding effect extends the lifetimes of two-dimensional sessile droplets. *J. Eng. Math.* **120**, 89–110 (2020).

61. Lohse, D. & Zhang, X. Physicochemical hydrodynamics of droplets out of equilibrium. *Nat. Rev. Phys.* **2**, 426–443 (2020).

62. Wray, A. W., Duffy, B. R. & Wilson, S. K. Competitive evaporation of multiple sessile droplets. *J. Fluid Mech.* **884**, A45 (2020).

63. Zhu, X., Verzicco, R., Zhang, X. & Lohse, D. Diffusive interaction of multiple surface nanobubbles: shrinkage, growth, and coarsening. *Soft Matter* **14**, 2006–2014 (2018).

64. Carrier, O. *et al.* Evaporation of water: evaporation rate and collective effects. *J. Fluid Mech.* **798**, 774–786 (2016).

65. Michelin, S., Guérin, E. & Lauga, E. Collective dissolution of microbubbles. *Phys. Rev. Fluids* **3**, 043601 (2018).
