# Supplementary material for: Substrate colonization by an emulsion drop prior to spreading
Source: Nat Commun. 2021 Sep 30;12:5734. doi: 10.1038/s41467-021-26015-2 (PMC8484436; doi:10.1038/s41467-021-26015-2)
Supplement: Supplementary file 3 — Description of Additional Supplementary Files [file 41467_2021_26015_MOESM3_ESM.pdf]

Description of Additional Supplementary Files

File Name: Supplementary Movie 1
Description: Evolution of interference patterns (left) when a glycerol drop (R=82 µm) in SO1000 settled under gravity, towards a native SU8 substrate (SO1000-G-NS) and the corresponding reconstructed film shapes (right). Scale bar: 10 µm.

File Name: Supplementary Movie 2
Description: Nucleated sites were observed only underneath the glycerol drop (SO1000-G-M). The video was captured 12 h after the onset of nucleation. Scale bar: 20 µm.

File Name: Supplementary Movie 3
Description: Long time growth dynamics of glycerol islands on mica underneath a glycerol drop (SO1000-G-M; R=126 µm). Scale bar: 10 µm.

File Name: Supplementary Movie 4
Description: Long time growth dynamics of glycerol islands on plasma-treated SU8 underneath a glycerol drop (SO1000-G-PS; R=88 µm). Scale bar: 10 µm

File Name: Supplementary Movie 5
Description: Instantaneous film rupture observed when a glycerol drop (R=35 µm) in castor oil meets the glycerol islands nucleated on a plasma-treated SU8 substrate. h_{min} in the video is the minimum film height at a given instant of time, obtained from the reconstructed film shapes. Scale bar: 10 µm.

File Name: Supplementary Movie 6
Description: A water drop (R=248 µm) at pH 5 settling in silicone oil (1000 cP) towards a plasma-treated SU8 substrate. This is to show that interfacial charging is not the reason for hindered coalescence between the parent drop and the islands. Scale bar: 10 µm.

File Name: Supplementary Movie 7
Description: Dehydrating the silicone can cause strong effects of polymer confinement, which led to islands pushing the parent glycerol drop upwards (left; SO1000-G-PS; R=107 µm). The presence of trace amounts of dissolved water eliminates this effect, and led to an instantaneous film rupture, upon contact between the parent drop and the island (right; SO1000-G-PS; R=108 µm). Scale bar: 10 µm.

File Name: Supplementary Movie 8
Description: Glycerol drop (R=103 µm) settling in castor oil predissolved with Span 80 (concentration=1.25 times CMC) towards plasma-treated SU8. Scale bar: 5 µm.

File Name: Supplementary Movie 9
Description: Glycerol drop (R=68 µm) settling in castor oil predissolved with Span 80 (concentration=4 times CMC) towards plasma-treated SU8. Scale bar: 5 µm.

File Name: Supplementary Movie 10
Description: Coalescence and relaxation of the final island to measure the viscosity of the immobilized polymer layer. Scale bar: 5 µm.
